# Diagnostic biomarkers and aortic dissection: a systematic review and meta-analysis

**DOI:** 10.1186/s12872-023-03448-9

**Published:** 2023-10-10

**Authors:** Hongjian Chen, Yunjie Li, Zheqian Li, Yanli Shi, Haobo Zhu

**Affiliations:** 1 Department of Infection Disease, Children’s Hospital of Nanjing Medical University, Nanjing, Jiangsu China; 2 Department of Pediatrics, The First Affiliated Hospital of Nanjing Medical University, Nanjing, Jiangsu China; 3 Department of Clinical Laboratory, Children’s Hospital of Nanjing Medical University, Nanjing, Jiangsu China; 4 Department of Urology, Children’s Hospital of Nanjing Medical University, Nanjing, Jiangsu China

**Keywords:** Aortic dissection, Diagnosis, Biomarkers, Systematic review, microRNA

## Abstract

**Background:**

Aortic dissection (AD) is a serious and fatal vascular disease. The earlier the condition of AD patients can be assessed precisely, the more scientifically controlled the patient’s condition will be. Therefore, timely and accurate diagnosis is significant for AD. Blood biomarker testing as a method of liquid biopsy can improve the diagnostic efficiency of AD. This study conducted a systematic review of the current blood diagnostic biomarkers of AD.

**Methods:**

The PubMed, Cochrane Library, Web of Science, and Embase electronic databases were systematically searched from inception to January 1, 2023, using the terms “aortic dissection”, “serum”, “plasma” and “diagnosis”. Stata 12.0 software was used to perform Random effects meta-analysis was performed using Stata 12.0 software to determine the effect sizes and corresponding 95% confidence intervals. Then, a summary receiver operator characteristic (SROC) curve was drawn, and the area under the ROC curve (AUC) was calculated.

**Results:**

D-dimer had the best sensitivity and AUC for AD, with values of 0.96 (95% CI: 0.93–0.98) and 0.95 (95% CI: 0.93–0.97), respectively. The sensitivity and AUC values for D-dimer with a cut-off value of 500 ng/mL were 0.97 (95% CI: 0.95–0.99) and 0.94 (95% CI: 0.92–0.96), respectively. In contrast, microRNA had a better specificity value for AD, at 0.79 (95% CI: 0.73–0.83).

**Conclusions:**

D-dimer and microRNA have good accuracy in the diagnosis of AD, but the specificity of D-dimer is worse, and studies of microRNA are insufficient. The combination of different biomarkers can improve the diagnostic accuracy. Other blood biomarkers are related to the pathological progression of AD and can be selected according to pathological progress.

**Supplementary Information:**

The online version contains supplementary material available at 10.1186/s12872-023-03448-9.

## Introduction

Aortic dissection (AD) is a serious and fatal vascular disease characterized by tearing of the intima of the aortic wall. Blood in the vascular lumen gradually enters the middle of the aortic wall, forms a pressure haematoma, rapidly spreads and expands along the long axis of the aorta, and eventually forms a dissection haematoma and enlarges the true and false lumen. Complications such as tamponade, aortic insufficiency, and poor perfusion occur when aortic collaterals are involved [1–5]. The incidence of AD is 3.5-6/100,000/year. If AD patients are not treated in a timely manner, approximately 24% will die within the first 24 h after the onset of symptoms, and 50% will die within 48 h after the onset of symptoms [6–8]. The clinical features of AD are diverse; however, some patients with atypical manifestations are misdiagnosed with limb ischaemia, abdominal pain, painless poor perfusion, and dyspnoea, or they go undiagnosed, which may prolong the diagnosis time of AD patients [1, 9, 10]. Aortic dissection, especially the accurate and timely diagnosis of acute aortic dissection, is significant for the results of drug and surgical treatment; the sooner the condition of patients can be accurately diagnosed, the more precisely controlled the condition of patients can be [11–13]. Currently, the diagnosis of AD requires image review, such as computed tomography (CT), transesophageal echocardiography (TEE) or magnetic resonance imaging (MRI); however, the usage of image review is based on clinical symptoms. Atypical clinical symptoms of AD may influence the usage of image review, and each AD patient may experience delays in the availability of image review. Additionally, whether image review is used may be limited by issues such as imaging cost and/or imaging availability [10, 14].

As a method of liquid biopsy, noninvasive and cost-effective blood biomarker testing has diagnostic accuracy for diseases, and the ability to distinguish between patients and nonpatients has also attracted an increasing amount of attention [15–19]. Likewise, blood diagnostic biomarkers in aortic dissection have received an increasing amount of attention in recent years. Therefore, specific blood diagnostic biomarkers in aortic dissection are needed in the clinical diagnosis, which can distinguish AD patients from non-AD patients, especially when distinguishing AD patients with atypical manifestations from non-AD patients. Blood diagnostic biomarkers can be used as a reliable diagnostic method to compensate for the lack of imaging examinations.

In conclusion, this study systematically reviews existing research on blood diagnostic biomarkers for AD and summarizes the advantages and disadvantages of each blood biomarker for diagnosis.

## Method

### Search strategy

**Fig. 1:**
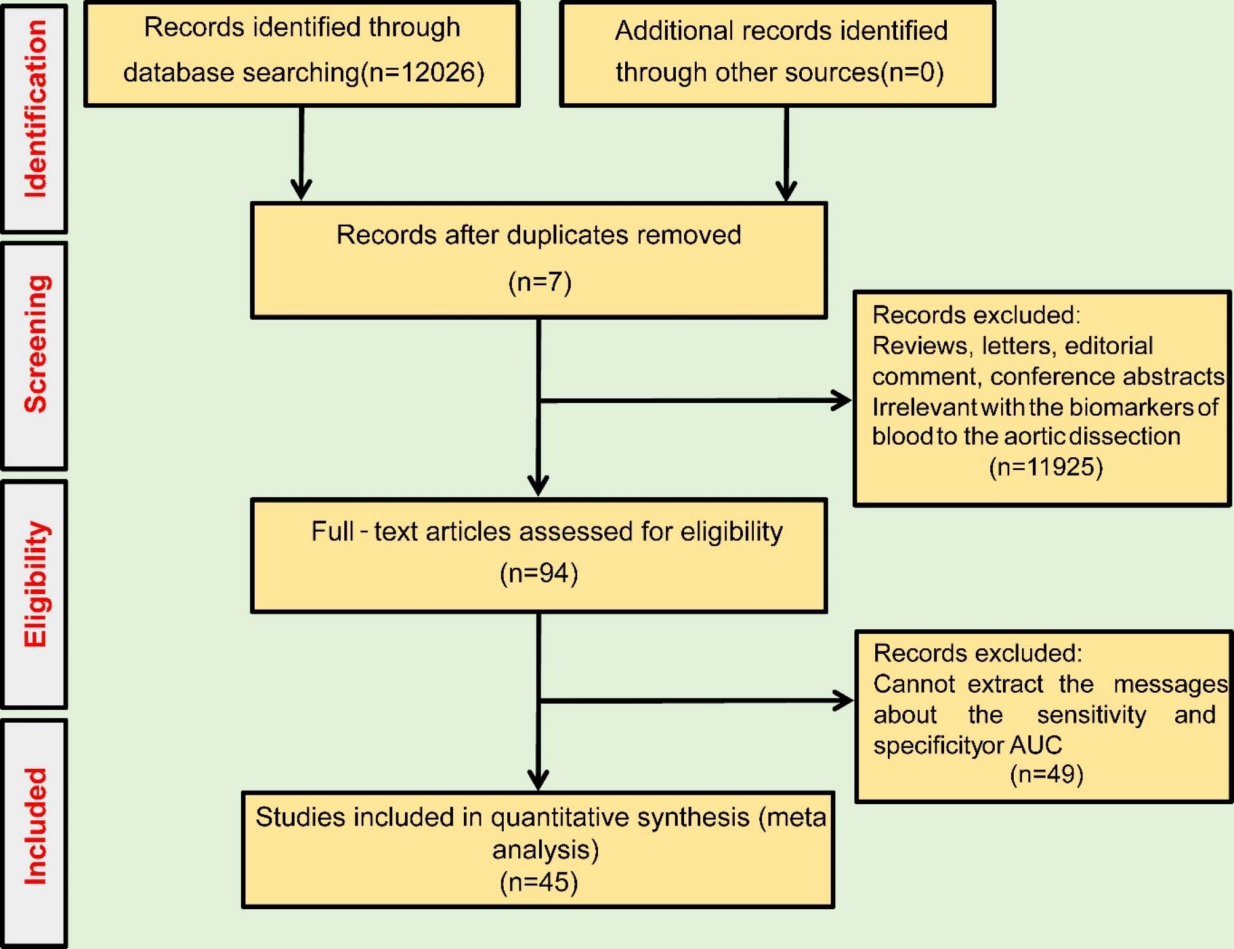
Flow chart of research selection in the review

The PubMed, Cochrane Library, Web of Science, and Embase electronic databases were systematically searched from inception to January 1, 2023, using MeSH terms such as “aortic dissection”, “serum”, “plasma” and “diagnosis”. Additionally, eligible studies were manually searched to ensure the comprehensiveness of the search. A total of 12,026 studies were initially retrieved. After excluding duplicate studies and screening studies according to the inclusion and exclusion criteria, 90 studies remained. Finally, studies that did not include data for extracting the area under the curve (AUC), diagnostic sensitivity and specificity values were excluded. Ultimately, a total of 46 studies were included in this systematic review (Fig. 1).

### Study selection

The literature included in this analysis met the following criteria: (1) the studies analysed the relationship between AD and blood diagnostic biomarkers; (2) the studies provided sensitivity and specificity values or AUC values, at least one of which could be obtained from the study; and (3) the studies were population-based studies. The exclusion criteria were as follows: (1) duplicate studies; (2) reviews, editorials, letters, conference abstracts; (3) studies with missing data; and (4) non-English studies. If the same author was included in different studies and the findings were from overlapping populations, only the first published study or the most complete study was included. The Quality Assessment of Diagnostic Accuracy Studies-2 (QUADAS-2) criteria were used to assess the quality of each included study.

### Data extraction

The following data were extracted from each study: first author name, publication year, type of disease, name of biomarker, size of patient population and nonpatient population, sample type, sensitivity, specificity, AUC, cut-off value and expression of ncRNA.

### Statistical analysis

Statistical analysis was performed by Stata 12.0 software. The effects and 95% confidence intervals (95% CI) were performed by a random effects model [20], including sensitivity, specificity, diagnostic odds ratio (DOR) and their 95% CI. The summary receiver operator characteristics (SROC) curve and the area under the curve of SROC (AUC) were used to assess the overall performance of the diagnostic test, and P < 0.05 (two-sided) was considered to indicate a statistically significant difference. Heterogeneity of study statistics was assessed by using the Q statistic and I^2^, where I^2^ > 50% indicated significant heterogeneity. Publication bias was assessed by using Deeks’ funnel plot.

## Results

### Literature search

According to the search results in Fig. 1, there were 25 and 5 papers analysing the diagnostic accuracy of D-dimer and noncoding RNA (ncRNA) for AD, respectively, which summarized the diagnostic effect value and the diagnostic effect of the other biomarkers for AD. Extracting the information according to the Data Extraction part of the method and the results are shown in Tables 1, 2, 3 and 4. The quality of the included studies was regarded as high according to the QUADAS-2 tool (Supplement Fig. 1).

**Table 1:** Characteristics of studies about D-dimer included in the analysis

No	Author	Year	Sample	Disease	Biomarker	AUC	Sen	Spe	Youden index	No. Case	No. Control	Cut-off value (ng/mL)	References
1	Forrer	2021	Plasma	AAD	D-dimer	0.7600	0.9700	0.3800	0.3500	34	150	300	[22]
2	Yang	2020	Plasma	TAD	D-dimer	0.8770	0.9850	0.6670	0.6520	78	72	NP	[23]
3	Wang	2018	Plasma	AAD	D-dimer	0.9100	0.8800	0.9400	0.8200	144	219	NP	[25]
4	Dong	2017	Plasma	AAD	D-dimer	0.6470	0.8110	0.4750	0.2860	37	40	NP	[26]
5	Li	2017	Plasma	AAD	D-dimer	0.9000	0.9400	0.5680	0.5080	202	588	500	[27]
6	Xiao	2016	Serum	AAD	D-dimer	0.8910	0.9333	0.6833	0.6166	60	60	1435	[28]
7	Gorla	2017	Plasma	AAD	D-dimer	0.9100	0.9600	0.6320	0.5920	376	291	500	[29]
8	Gorla	2017	Plasma	AAD	D-dimer	0.9600	0.9900	0.6700	0.6600	159	72	500	[30]
9	Yoshimuta	2015	Plasma	AAD	D-dimer	NP	1.0000	0.9480	0.9480	9	1227	6900	[31]
10	Peng	2015	Serum	AAD	D-dimer	0.9300	0.8000	0.9021	0.7021	35	52	2110	[32]
11	Shao	2014	Plasma	AAD	D-dimer	0.8080	0.6970	0.7740	0.4710	89	279	320	[44]
12	Nazerian	2014	Venous blood	AAD	D-dimer	NP	0.9830	0.3590	0.3420	233	802	500	[40]
13	Okazaki	2014	Plasma	AAD	D-dimer	0.9000	0.8000	0.9780	0.7780	15	46	8700	[42]
14	Giachino	2013	Plasma	AAD	D-dimer	0.8700	0.9760	0.3280	0.3040	52	74	500	[37]
15	Ersel	2010	Serum	AAD	D-dimer	0.7640	0.9660	0.9730	0.9390	30	69	NP	[35]
16	Fan	2010	Plasma	AAD	D-dimer	0.8950	0.9910	0.4180	0.4090	107	136	260	[36]
17	Sbarouni	2007	Plasma	AAD	D-dimer	NP	0.9400	0.5900	0.5300	18	29	700	[43]
18	Ohlmann	2006	Plasma	AAD	D-dimer	0.8800	0.9900	0.3400	0.3300	94	94	400	[41]
19	Hazui	2005	Plasma	AAD	D-dimer	0.9780	0.9310	0.9310	0.8620	29	49	800	[38]
20	Akutsu	2005	Plasma	AAD	D-dimer	NP	1.0000	0.5400	0.5400	30	48	500	[33]
21	Eggebrecht	2004	Plasma	AAD	D-dimer	0.8650	1.0000	0.7300	0.7300	16	48	626	[34]
22	Weber	2003	NP	AAD	D-dimer	NP	1.0000	0.6857	0.6857	24	35	500	[45]

AUC, area under the curve of receiver operator characteristics. Sen, sensitivity. Spe, specificity. AAD, acute aortic dissection. AD, aortic dissection. NP, not report

**Table 2:** Characteristics of studies about combined D-dimer and other biomarkers included in the analysis

No	Author	Year	Sample	Disease	Biomarker	AUC	Sen	Spe	Youden index	No. Case	No. Control	References
1	Yang	2020	Plasma	TAD	D-dimer + hs-CRP + ANGPTL8	0.9270	0.7950	0.9850	0.7800	78	72	[23]
2	Yang	2020	Plasma	TAD	D-dimer + ANGPTL8	0.9090	0.8330	0.8940	0.7270	78	72	[23]
3	Xiao	2016	Serum	AAD	Lumican + D-dimer	0.9620	0.8833	0.9500	0.8330	60	60	[28]
4	Giachino	2013	Plasma	AAD	log2MMP8 + D-dimer	0.8900	1.0000	0.1310	0.1310	52	74	[37]
5	He	2019	Plasma	AAD	SAA + D-dimer	0.9000	0.8480	0.9380	0.7860	63	87	[39]
6	Ma	2021	Serum	AAD	CP + D-dimer	0.7070	0.6360	0.9410	0.5770	102	85	[21]

AUC, area under the curve of receiver operator characteristics. Sen, sensitivity. Spe, specificity. AAD, acute aortic dissection

**Table 3:** Characteristics of studies about microRNA included in the analysis

No	Author	Year	Sample	Type of disease	Biomarker	AUC	Sen	Spe	Youden index	No. Case	No. Control	References
1	Dong	2017	Plasma	AAD	miR-15a	0.7610	0.7570	0.8250	0.582	37	40	[26]
2	Dong	2017	Plasma	AAD	miR-23a	0.7340	0.8650	0.6250	0.490	37	40	[26]
3	Dong	2017	Plasma	AAD	let-7b	0.7290	0.7940	0.6920	0.486	37	40	[26]
4	Dong	2017	Plasma	AAD	hcmv-miR-US-33-5p	0.6570	0.7350	0.6410	0.376	37	40	[26]
5	Wang	2017	Plasma	AAD	miR-4787-5p	0.8980	NP	NP	NP	98	56	[24]
6	Wang	2017	Plasma	AAD	miR-4306	0.8740	NP	NP	NP	98	56	[24]
7	Tian	2019	Serum	AAAD	circMARK3	0.9344	0.9000	0.8670	0.767	30	30	[46]
8	Senturk	2019	Serum	TAD	hsa-miR-143-3p	0.6000	NP	NP	NP	9	10	[47]
9	Senturk	2019	Serum	TAD	hsa-miR-22-3p	0.5000	NP	NP	NP	9	10	[47]
10	Xu	2017	Serum	AAAD	miR-26b	0.9110	0.8800	0.9000	0.780	25	30	[48]
11	Xu	2017	Serum	AAAD	miR-29a	0.8990	0.8000	0.9333	0.733	25	30	[48]
12	Xu	2017	Serum	AAAD	miR-25	0.8810	0.9200	0.7667	0.687	25	30	[48]
13	Xu	2017	Serum	AAAD	miR-155	0.8630	0.8400	0.8333	0.673	25	30	[48]
14	Xu	2017	Serum	AAAD	miR-29a	0.8970	0.7813	0.8621	0.643	64	58	[48]
15	Xu	2017	Serum	AAAD	miR-155	0.8710	0.8438	0.7759	0.620	64	58	[48]
16	Xu	2017	Serum	AAAD	miR-25	0.8570	0.8125	0.7414	0.554	64	58	[48]
17	Xu	2017	Serum	AAAD	miR-26b	0.8030	0.6563	0.8276	0.484	64	58	[48]

AUC, area under the curve of receiver operator characteristics. Sen, sensitivity. Spe, specificity. AAD, acute aortic dissection. AD, aortic dissection. NP, not report

**Table 4:** Characteristics of studies about combined ncRNA included in the analysis

No	Author	Year	Sample	Type of disease	Biomarker	AUC	Sen	Spe	Youden index	No. Case	No. Control	References
1	Wang	2017	Plasma	AAD	miR-4787-5p + miR-4306	0.9610	NP	NP	NP	98	56	[27]
2	Tian	2019	Serum	AAAD	circMARK3 + miR-1273-3p	0.9644	0.9330	0.8670	0.800	30	30	[46]
3	Xu	2017	Serum	AAAD	miR-26b + miR-29a + miR-25 + miR-155	0.9950	0.9600	1.0000	0.960	25	30	[48]
4	Xu	2017	Serum	AAAD	miR-26b + miR-29a + miR-25 + miR-155	0.9780	0.8906	0.9483	0.839	64	58	[48]

AUC, area under the curve of receiver operator characteristics. Sen, sensitivity. Spe, specificity. AAAD, acute type A aortic dissection. AAD, acute aortic dissection. NP, not report

### Meta-analysis of the diagnostic accuracy of D-dimer for AD

**Fig. 2:**
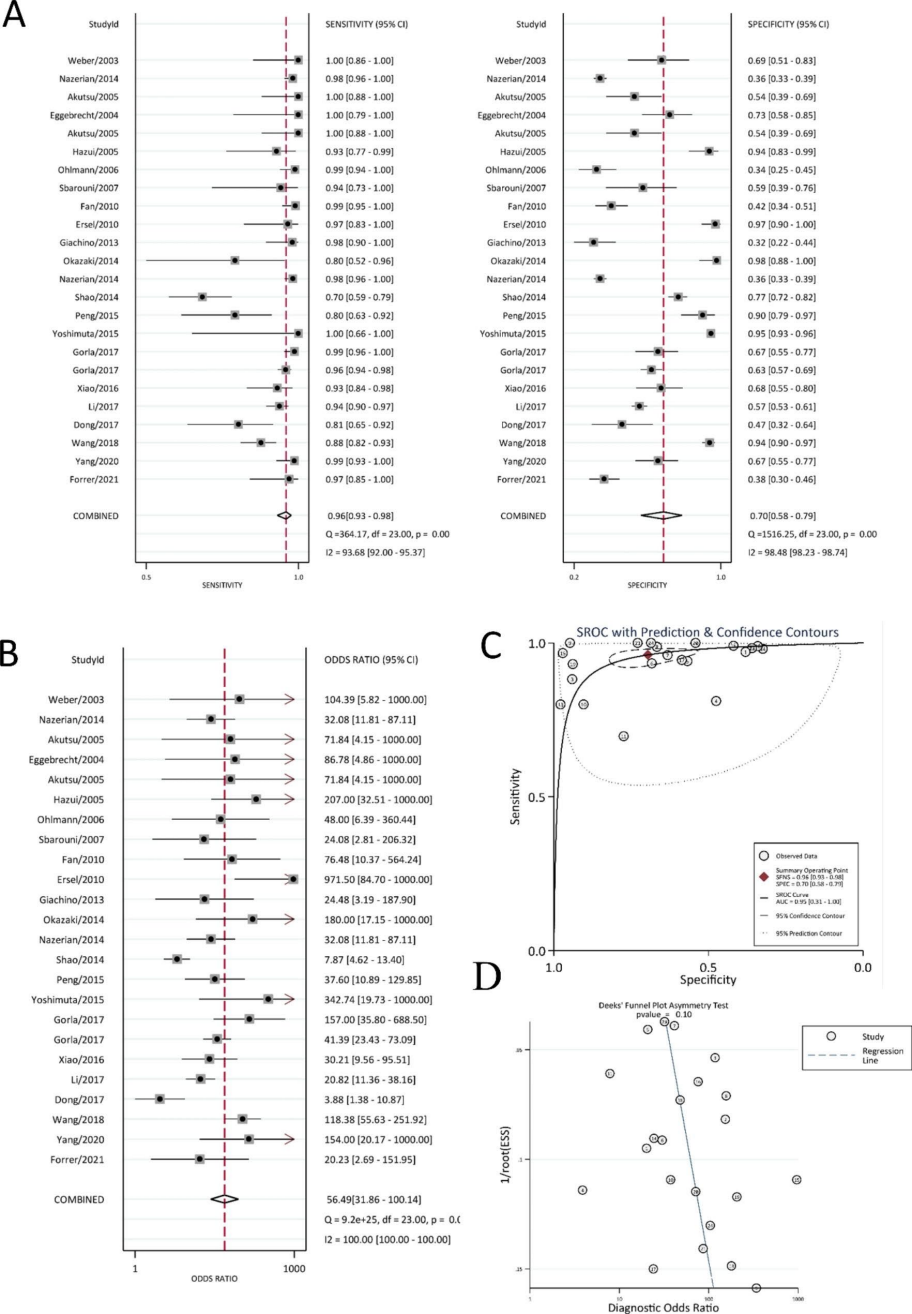
Diagnostic accuracy for D-dimer (**A**) Diagnostic sensitivity and specificity (**B**) Diagnostic accuracy (**C**) Receiver operating characteristic curve (ROC) (**D**) Publication bias

**Fig. 3:**
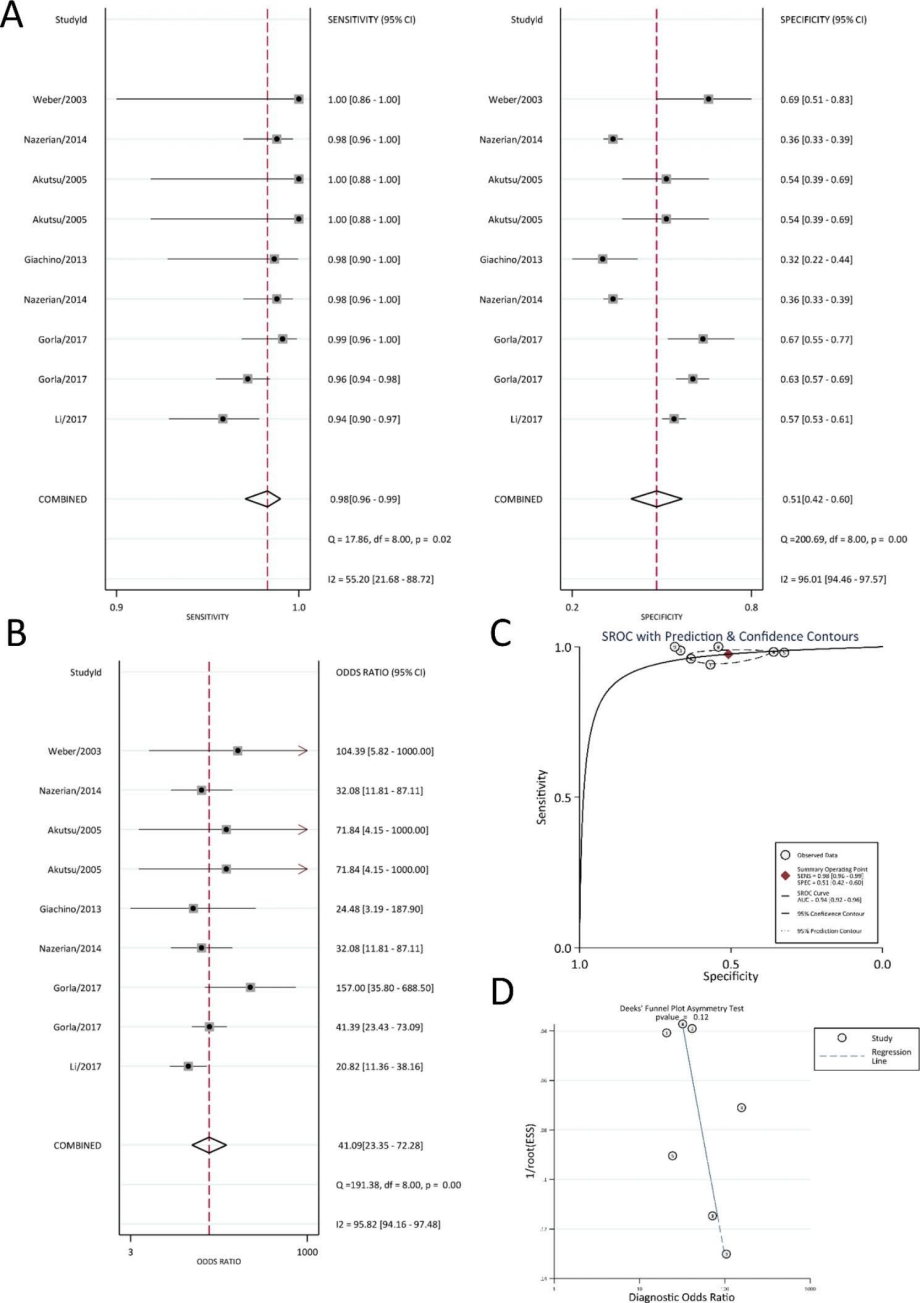
Diagnostic accuracy for D-dimer of 500ng/mL cut-off value (**A**) Diagnostic sensitivity and specificity (**B**) Diagnostic accuracy (**C**) Receiver operating characteristic curve (ROC) (**D**) Publication bias

A total of 12,026 records were initially identified through systematic searches of the electronic databases, of which 95 records were retrieved for full-text review, and 24 papers [21–45] and 30 studies on the diagnostic accuracy of D-dimer for AD were eligible based on the inclusion and exclusion criteria (Tables 1 and 2). The random effects model was used to perform the meta-analysis [20]. The results are shown in Figs. 2 and 3. For the accuracy of D-dimer in diagnosing aortic dissection, the summary sensitivity, specificity and DOR values were 0.96 (95% CI: 0.93–0.98), 0.72 (95% CI: 0.59–0.81), and 56.86 (95% CI: 30.87-104.72), respectively (Fig. 2A-B), and the AUC was 0.95 (95% CI: 0.93–0.97) (Fig. 2C). Deeks’ funnel plot (Fig. 2D) showed that there was no publication bias in the included literature. Subgroup analysis of studies [21, 23, 28, 37, 39] with a D-dimer cut-off value of 500 ng/mL was performed. The summary sensitivity, specificity and DOR values were 0.97 (95% CI: 0.95–0.99), 0.53 (95% CI: 0.43–0.63), and 41.58 (95% CI: 21.52–80.32), respectively, the AUC was 0.94 (95% CI: 0.92–0.96) (Fig. 3A-C).

According to the results, the sensitivity of D-dimer for the diagnostic accuracy of AD was good, and the summary sensitivity value was 0.96, but the specificity of D-dimer for the diagnosis of AD was poor, and the summary specificity value was 0.72. The summary specificity value dropped to 0.53 after subgroup analysis with a D-dimer cut-off value of 500 ng/ml. Additionally, there was research on the diagnostic accuracy of D-dimer combined with other biomarkers for AD (Table 2). The results showed that compared with testing D-dimer only, the combination with other biomarkers can significantly improve the specificity, while the AUC and sensitivity values remained above 0.8. According to the data in Table 1, the AUC value of D-dimer for the diagnostic accuracy of AD ranged from 0.6 to 1.

Due to the observed high heterogeneity in the results of the meta-analysis, we conducted a meta-regression analysis to investigate the potential sources of heterogeneity. Specifically, we examined the publication year (before 2017 or after 2017), sample size (greater than 100 or less than 100), use of 500 ng/mL as a cut-off value, and geographical location of the study population (Asian or non-Asian) (see Supplementary Fig. 1). Additionally, we performed subgroup meta-analyses based on the publication year, sample size, and geographical location (Supplementary Figs. 2–7). However, these analyses did not reveal a significant reduction in heterogeneity.

### Meta-analysis of the diagnostic accuracy of noncoding RNAs for AD

**Fig. 4:**
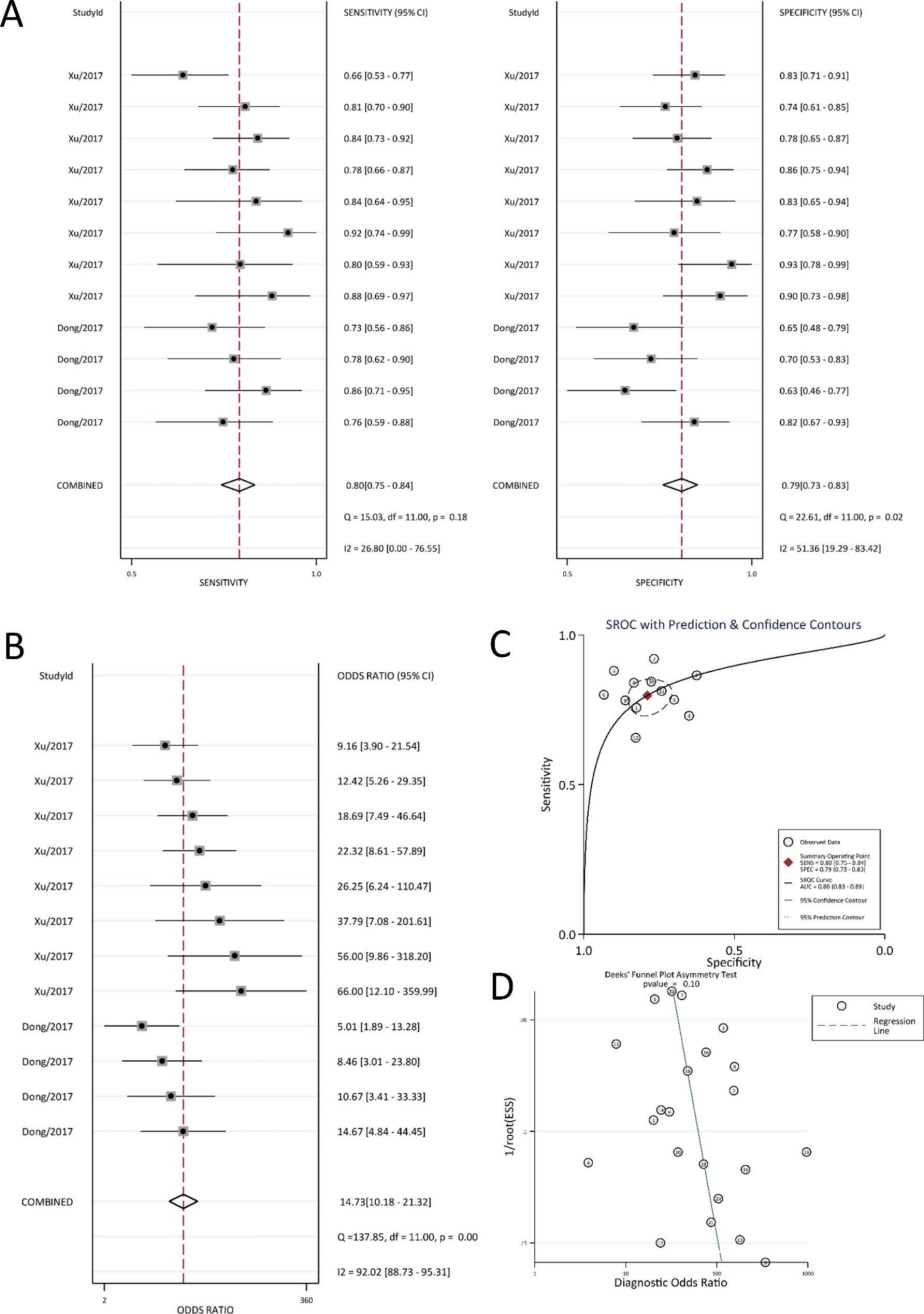
Diagnostic accuracy for microRNA (**A**) Diagnostic sensitivity and specificity (**B**) Diagnostic accuracy (**C**) Receiver operating characteristic curve (ROC) (**D**) Publication bias

A total of 12,026 records were identified through systematic searches in electronic databases. Of these, 95 records were retrieved for full-text review, and 5 papers [24, 26, 46–48] and 21 studies on the diagnostic accuracy of ncRNA for AD were eligible based on the inclusion and exclusion criteria (Tables 3 and 4). Sixteen of the 17 studies analysed the diagnostic accuracy of microRNAs for AD, as shown in Table 3; hence, we performed a meta-analysis of microRNAs. The random effects model was used to perform the meta-analysis [20]. The results are shown in Fig. 4. For the accuracy of ncRNA in diagnosing AD, the summary sensitivity, specificity, and DOR values were 0.80 (95% CI: 0.75–0.84), 0.79 (95% CI: 0.73–0.83), and 14.73 (95% CI: 10.86–23.78), respectively (Fig. 4A-B), and the AUC was 0.86 (95% CI: 0.83–0.89) (Fig. 4C). Deeks’ funnel plot (Fig. 4D) showed no publication bias in the included literature, which indicated that the sensitivity and specificity of microRNA for the diagnostic accuracy of AD were good.

Additionally, some papers [27, 46, 48] reported the diagnostic accuracy of the combination of two ncRNAs, including microRNA and circular RNA (circRNA) (Table 4). The results showed that the combination of ncRNAs can significantly improve the diagnostic accuracy of AD, such as testing miR-26b, miR-29a, miR-25 and miR-155 only for the diagnosis of AD, for which the AUC, sensitivity and specificity values, except the AUC value of miR-26b, which exceeded 0.9, and the specificity values of miR-25, which exceeded 0.9, were all less than 0.9. However, combining miR-26b, miR-29a, miR-25 and miR-155 improved the AUC, sensitivity and specificity values, sharply enhancing the diagnostic accuracy of ncRNAs for AD.

It is important to highlight that there could potentially be an issue concerning the accessibility of microRNA. In addition, it is crucial to consider the significance of the limited accessibility of these biological indicators, as delays in intervention may potentially correlate with unfavorable outcomes in AD.

### Other diagnostic biomarkers of AD

A total of 18 papers were included in this study, and there were 55 studies on the diagnostic accuracy of other biomarkers (excluding D-dimer and ncRNA) for AD (Supplementary Table 1). The following criteria were used to filter studies: AUC value greater than 0.9 and Youden index greater than 0.8. Studies No. 1–13 were selected to further describe their possible roles in AD pathological changes. The biomarkers involved were osteopontin (OPN), ADAMTS1, ADAMTS4, soluble ST2 (sST2), aggrecan (ACAN), serum amyloid A (SAA), ceruloplasmin (CP), polycystin 1 (PC1), and monocyte-to-high-density lipoprotein ratio (MHR) (Supplementary Table 2). Supplementary Tables 3 and Supplementary Table 4 summarize the dysregulated miRNAs, lncRNAs and circRNAs and their roles in AD. Supplementary Table 5 summarizes other systematic reviews of the diagnostic accuracy of D-dimer in AD.

## Discussion

This study included 45 papers on blood diagnostic biomarkers for AD and systematically evaluated their role in AD diagnosis. D-dimer had the best diagnostic accuracy for AD among them, and the sensitivity of subgroup analysis of D-dimer with a cut-off value of 500 ng/mL had the highest value of approximately 0.97 (95% CI: 0.95–0.99). The diagnostic accuracy of microRNA for AD was worse than that of D-dimer; however, its sensitivity, specificity and AUC values for AD were 0.80 (95% CI: 0.75–0.84), 0.79 (95% CI: 0.73–0.83) and 0.86 (95% CI: 0.83–0.89), respectively. The other studies included in this meta-analysis also examined the blood diagnostic biomarkers of AD. However, the studies were insufficient for analysis, and this study only focused on the possible mechanisms and functions of AD (Supplementary Table 2).

According to the criteria proposed by Jones [49], an AUC value greater than 0.97 was classified as “excellent”, an AUC value ranging from 0.93 to 0.96 was classified as “very good”, an AUC value ranging from 0.75 to 0.92 was classified as “good”, and an AUC value less than 0.75 was classified as “reasonable”. The Youden index, which ranges from 0 to 1, can be used to determine the best cut-off value of biomarkers to distinguish between patients and nonpatients [50]. When the Youden index is equal to 1, the biomarker can completely separate the patients and nonpatients, which cannot be separated when the Youden index is equal to 0; therefore, a higher Youden index indicates a better ability to distinguish between patients and nonpatients [51]. D-dimer had the highest AUC values, both greater than 0.95, which could be classified as “very good”. The sensitivity value of D-dimer for the diagnostic accuracy of AD was 0.96 (95% CI: 0.93–0.98), but the specificity value was only 0.72 (95% CI: 0.59–0.81), and the Youden index was 0.68. After subgroup analysis with a D-dimer cut-off value of 500 ng/mL, the AUC, sensitivity and DOR values did not change significantly; however, the specificity value dropped sharply to 0.53 (95% CI: 0.42–0.60), and the Youden index also dropped to 0.50. D-dimer with different cut-off values ​​had different diagnostic accuracies in clinical diagnosis. However, because of its low specificity, it may cause a large false-positive rate.

The AUC value of ncRNA was 0.86 (95% CI: 0.83–0.89), which could be classified as “good”. Compared with D-dimer, the specificity of microRNA was better; however, the sensitivity and Youden index were poorer, meaning that microRNA as a blood diagnostic biomarker of AD may be worse than that of D-dimer, but the specificity of microRNA can reduce the false-positive rate of AD. In conclusion, both D-dimer and microRNA have the potential to be used as blood diagnostic biomarkers of AD, and they can be used as blood diagnostic biomarkers of AD according to their different characteristics.

The combination of blood diagnostic biomarkers can improve the diagnostic accuracy of AD. Tables 2 and 4 summarize the studies on the diagnostic accuracy of AD using a combination of different blood diagnostic biomarkers. The combination of D-dimer and other biomarkers can greatly improve the specificity of AD diagnosis according to Table 2, which has little effect on the diagnostic sensitivity. According to Table 4, the combination of microRNA and microRNA or circRNA significantly increased the AUC, sensitivity and specificity and significantly improved the diagnostic accuracy of ncRNA of AD, suggesting that we can consider the combination of multiple biomarkers to improve the accuracy of diagnosis when performing research on blood diagnostic biomarkers of AD, such as D-dimer, ncRNA and other biomarkers.

The pathological development of AD is a multistage process involving changes in different biomarkers. D-dimer is a biomarker of coagulation and fibrinolytic system activation that can be detected and measured in whole blood or plasma and serves as an indirect marker of thrombotic activity [52–55]. The pathological development of AD is a multistage process involving changes in different biomarkers. D-dimer is a biomarker of coagulation and fibrinolytic system activation that can be detected and measured in whole blood or plasma and serves as an indirect marker of thrombotic activity [52–55]. Studies have found that D-dimer levels are low in the circulatory system in healthy people and are elevated in thrombotic diseases [56]. The activation of coagulation and fibrinolysis systems and thrombosis also exist in AD, meaning that D-dimer, as a biomarker of AD, can precisely detect the occurrence and development of AD.

NcRNAs are also involved in the pathological development of AD. NcRNAs include long noncoding RNAs (lncRNAs), circular RNAs (circRNAs) and microRNAs (miRNAs). The major pathological changes in AD are degeneration of the vessel medial wall, including phenotypic changes and reductions in vascular smooth muscle cells (VSMCs), elastin fragmentation and degeneration, extracellular matrix (ECM) degradation, and inflammatory cell infiltration. Studies have shown that lncRNAs, circRNAs and miRNAs are involved in the pathological development of AD in Supplementary Materials, including miR-21 in VSMCs, which can promote phenotypic transition by targeting SMAD7 [57], and lncRNA-XIST can inhibit cell proliferation through the miR-17/PTEN axis in VSMCs [58]. NcRNAs can influence biological processes through posttranscriptional regulation and are differentially expressed in two different states of cells or tissues; for example, miR-22-3p [47, 59] and miR-26b [48, 60] were downregulated in aortic tissue of AD cases and involved in the regulation of VSMC proliferation and apoptosis. CircMARK3 [46] was upregulated in AD tissue and had the same expression in the circulatory system of AD patients. The AUC values of miR-22-3p, miR-26b and circMARK3 were 0.5, 0.911, 0.8030 and 0.9344, respectively (2 studies on the diagnostic accuracy of miR-26b), suggesting that the expression of ncRNA in AD tissues is the same as that in the circulatory system.

Regarding the other biomarkers examined herein, it was surprising that they have been verified to play an important role in the pathological progression of AD, such as the phenotypic transformation of VSMCs, proliferation, ECM dysfunction and infiltration of inflammatory cells. Although an AUC value greater than 0.9 and a Youden index greater than 0.8 were used as screening criteria to screen out 9 biomarkers for a more detailed description, the role of other biomarkers in Supplementary Table 1 was verified for the progression of AD, which was strikingly similar to the biomarkers in Supplementary Table 2. IL-6, IL-10 and IL-16 regulate blood vessels by upregulating MCP-1 and activating macrophage inflammation [61]. IL-10 is elevated in the plasma of AD patients [62]. IL-16 is a regulator of VSMC migration and invasion by binding to CD4 and inducing p38MAPK phosphorylation, MMP9 expression and Sp-1 binding activation [63].

Although the diagnostic accuracy of relevant biomarkers of AD has been revealed previously, based on our study, the studies to identify the blood biomarkers of AD diagnostics were still insufficient. The diagnostic accuracy of D-dimer for AD has been extensively studied, and D-dimer has been written into the “Chinese Expert Consensus on the Diagnosis and Treatment of Aortic Dissection” and has been applied to the diagnosis of clinical diseases, including AD [3]. However, the cut-off value of 500 ng/ml D-dimer did not show the optimal diagnostic effect, and a high-quality large-scale prospective cohort study is needed to determine the optimal cut-off value of D-dimer for the diagnosis of AD.

The present investigation exhibits various limitations. Different kinds of aortic dissection may have varying impacts on several biomarkers. However, the current body of literature lacks sufficient information to complete this subgroup analysis due to the limited inclusion of full descriptions pertaining to the many subtypes of aortic dissection patients in many publications. The present study solely investigated a limited number of other biomarkers that exhibited superior diagnostic efficacy, as described in the section dedicated to other biomarkers. However, a comprehensive analysis of several clinically relevant indicators, such as the white blood cell count, high sensitive troponin, interleukin 6 and 10, and plasma activator inhibitor 1, was not provided.

## Conclusion

When combining the AUC, sensitivity, specificity and Youden index for the diagnosis of AD, the data of the biomarkers were ideal, but the diagnostic accuracy of AD was insufficient, as only 1 study indicated the diagnostic accuracy of biomarkers for AD. Only relying on the data provided by 1 study to prove that a biomarker can be used as a blood diagnostic biomarker of AD is less rigorous. In conclusion, this systematic review can only prove that biomarkers may be used as blood biomarkers of AD.

### Consent to publish

Not applicable.

### Electronic supplementary material

Below is the link to the electronic supplementary material.

Supplementary Material 1

## Data Availability

The datasets supporting the conclusions of this article are included within the article. All data used in this study have been retrieved from publicly available published papers, so approval from the institutional review board was not needed.

## References

[1] Golledge J, Eagle KA (2008). Acute aortic dissection. Lancet (London England).

[2] Roberts CS, Roberts WC (1991). Aortic dissection with the entrance tear in the descending thoracic aorta. Analysis of 40 necropsy patients. Ann Surg.

[3] Roberts CS, Roberts WC (1990). Aortic dissection with the entrance tear in transverse aorta: analysis of 12 autopsy patients. Ann Thorac Surg.

[4] Pape LA, Awais M, Woznicki EM, Suzuki T, Trimarchi S, Evangelista A, Myrmel T, Larsen M, Harris KM, Greason K (2015). Presentation, diagnosis, and outcomes of Acute Aortic dissection: 17-Year Trends from the International Registry of Acute Aortic Dissection. J Am Coll Cardiol.

[5] Zhu Y, Lingala B, Baiocchi M, Tao JJ, Toro Arana V, Khoo JW, Williams KM, Traboulsi AA, Hammond HC, Lee AM (2020). Type a aortic dissection-experience over 5 decades: JACC historical breakthroughs in perspective. J Am Coll Cardiol.

[6] Erbel R, Aboyans V, Boileau C, Bossone E, Bartolomeo RD, Eggebrecht H, Evangelista A, Falk V, Frank H, Gaemperli O (2014). 2014 ESC Guidelines on the diagnosis and treatment of aortic diseases: document covering acute and chronic aortic diseases of the thoracic and abdominal aorta of the adult. The Task Force for the diagnosis and treatment of aortic Diseases of the European Society of Cardiology (ESC). Eur Heart J.

[7] Clouse WD, Jr Hallett JW, Schaff HV, Spittell PC, Rowland CM, Ilstrup DM, Melton LJ (2004). Acute aortic dissection: population-based incidence compared with degenerative aortic aneurysm rupture. Mayo Clin Proc.

[8] Howard DP, Banerjee A, Fairhead JF, Perkins J, Silver LE, Rothwell PM (2013). Population-based study of incidence and outcome of acute aortic dissection and premorbid risk factor control: 10-year results from the Oxford Vascular Study. Circulation.

[9] Rapezzi C, Longhi S, Graziosi M, Biagini E, Terzi F, Cooke RM, Quarta C, Sangiorgi D, Ciliberti P, Di Pasquale G (2008). Risk factors for diagnostic delay in acute aortic dissection. Am J Cardiol.

[10] Ranasinghe AM, Bonser RS (2010). Biomarkers in acute aortic dissection and other aortic syndromes. J Am Coll Cardiol.

[11] Hagan PG, Nienaber CA, Isselbacher EM, Bruckman D, Karavite DJ, Russman PL, Evangelista A, Fattori R, Suzuki T, Oh JK (2000). The International Registry of Acute Aortic dissection (IRAD): new insights into an old disease. JAMA.

[12] Januzzi JL, Isselbacher EM, Fattori R, Cooper JV, Smith DE, Fang J, Eagle KA, Mehta RH, Nienaber CA, Pape LA (2004). Characterizing the young patient with aortic dissection: results from the International Registry of aortic dissection (IRAD). J Am Coll Cardiol.

[13] Nienaber CA, Eagle KA (2003). Aortic dissection: new frontiers in diagnosis and management: part I: from etiology to diagnostic strategies. Circulation.

[14] Suzuki T, Distante A, Eagle K (2010). Biomarker-assisted diagnosis of acute aortic dissection: how far we have come and what to expect. Curr Opin Cardiol.

[15] Siravegna G, Marsoni S, Siena S, Bardelli A (2017). Integrating liquid biopsies into the management of cancer. Nat reviews Clin Oncol.

[16] Russano M, Napolitano A, Ribelli G, Iuliani M, Simonetti S, Citarella F, Pantano F, Dell’Aquila E, Anesi C, Silvestris N (2020). Liquid biopsy and tumor heterogeneity in metastatic solid tumors: the potentiality of blood samples. J experimental Clin cancer research: CR.

[17] Best MG, Sol N, Zijl S, Reijneveld JC, Wesseling P, Wurdinger T (2015). Liquid biopsies in patients with diffuse glioma. Acta Neuropathol.

[18] Geeurickx E, Hendrix A (2020). Targets, pitfalls and reference materials for liquid biopsy tests in cancer diagnostics. Mol Aspects Med.

[19] Di Meo A, Bartlett J, Cheng Y, Pasic MD, Yousef GM (2017). Liquid biopsy: a step forward towards precision medicine in urologic malignancies. Mol Cancer.

[20] Borenstein M, Hedges LV, Higgins JP, Rothstein HR (2010). A basic introduction to fixed-effect and random-effects models for meta-analysis. Res synthesis methods.

[21] Ma C, Zhao H, Shi F, Li M, Liu X, Ji C, Han Y (2021). Serum ceruloplasmin is the candidate predictive biomarker for Acute Aortic dissection and is related to Thrombosed false lumen: a Propensity score-matched observational case-control study. Biol Trace Elem Res.

[22] Forrer A, Schoenrath F, Torzewski M, Schmid J, Franke UFW, Göbel N, Aujesky D, Matter CM, Lüscher TF, Mach F et al. Novel blood biomarkers for a diagnostic workup of Acute Aortic Dissection. Diagnostics (Basel Switzerland) 2021, 11(4). 10.3390/diagnostics11040615 PMC8065878 33808169

[23] Yang Y, Jiao X, Li L, Hu C, Zhang X, Pan L, Yu H, Li J, Chen D, Du J (2020). Increased circulating Angiopoietin-Like protein 8 levels are Associated with thoracic aortic dissection and higher inflammatory conditions. Cardiovasc Drugs Ther.

[24] Wang L, Zhang S, Xu Z, Zhang J, Li L, Zhao G. The diagnostic value of microRNA-4787-5p and microRNA-4306 in patients with acute aortic dissection. Am J translational Res 2017, 9(11):5138–49. PMC5714797 29218111

[25] Wang Y, Tan X, Gao H, Yuan H, Hu R, Jia L, Zhu J, Sun L, Zhang H, Huang L (2018). Magnitude of Soluble ST2 as a Novel Biomarker for Acute Aortic Dissection. Circulation.

[26] Dong J, Bao J, Feng R, Zhao Z, Lu Q, Wang G, Li H, Su D, Zhou J, Jing Q (2017). Circulating microRNAs: a novel potential biomarker for diagnosing acute aortic dissection. Sci Rep.

[27] Li W, Huang B, Tian L, Yang Y, Zhang W, Wang X, Chen J, Sun K, Hui R, Fan X (2017). Admission D-dimer testing for differentiating acute aortic dissection from other causes of acute chest pain. Archives of medical science: AMS.

[28] Xiao Z, Xue Y, Yao C, Gu G, Zhang Y, Zhang J, Fan F, Luan X, Deng Z, Tao Z (2016). Acute aortic dissection biomarkers identified using Isobaric Tags for relative and absolute quantitation. Biomed Res Int.

[29] Gorla R, Erbel R, Kahlert P, Tsagakis K, Jakob H, Mahabadi AA, Schlosser T, Eggebrecht H, Bossone E, Jánosi RA (2017). Accuracy of a diagnostic strategy combining aortic dissection detection risk score and D-dimer levels in patients with suspected acute aortic syndrome. Eur heart J Acute Cardiovasc care.

[30] Gorla R, Erbel R, Kahlert P, Tsagakis K, Jakob H, Mahabadi AA, Schlosser T, Eggebrecht H, Bossone E, Jánosi RA (2017). Diagnostic role and prognostic implications of D-dimer in different classes of acute aortic syndromes. Eur heart J Acute Cardiovasc care.

[31] Yoshimuta T, Yokoyama H, Okajima T, Tanaka H, Toyoda K, Nagatsuka K, Higashi M, Hayashi K, Kawashiri MA, Yasuda S (2015). Impact of elevated D-Dimer on diagnosis of Acute Aortic Dissection with isolated neurological symptoms in ischemic stroke. Circulation journal: official journal of the Japanese Circulation Society.

[32] Peng W, Peng Z, Chai X, Zhu Q, Yang G, Zhao Q, Zhou S (2015). Potential biomarkers for early diagnosis of acute aortic dissection. Heart & lung: the journal of critical care.

[33] Akutsu K, Sato N, Yamamoto T, Morita N, Takagi H, Fujita N, Tanaka K, Takano T (2005). A rapid bedside D-dimer assay (cardiac D-dimer) for screening of clinically suspected acute aortic dissection. Circulation journal: official journal of the Japanese Circulation Society.

[34] Eggebrecht H, Naber CK, Bruch C, Kröger K, von Birgelen C, Schmermund A, Wichert M, Bartel T, Mann K, Erbel R (2004). Value of plasma fibrin D-dimers for detection of acute aortic dissection. J Am Coll Cardiol.

[35] Ersel M, Aksay E, Kıyan S, Bayraktaroğlu S, Yürüktümen A, Ozsaraç M, Calkavur T (2010). Can D-dimer testing help emergency department physicians to detect acute aortic dissections? *Anadolu kardiyoloji dergisi: AKD = the*. Anatol J Cardiol.

[36] Fan QK, Wang WW, Zhang ZL, Liu ZJ, Yang J, Zhao GS, Cao SZ (2010). Evaluation of D-dimer in the diagnosis of suspected aortic dissection. Clin Chem Lab Med.

[37] Giachino F, Loiacono M, Lucchiari M, Manzo M, Battista S, Saglio E, Lupia E, Moiraghi C, Hirsch E, Mengozzi G (2013). Rule out of acute aortic dissection with plasma matrix metalloproteinase 8 in the emergency department. Crit Care (London England).

[38] Hazui H, Fukumoto H, Negoro N, Hoshiga M, Muraoka H, Nishimoto M, Morita H, Hanafusa T (2005). Simple and useful tests for discriminating between acute aortic dissection of the ascending aorta and acute myocardial infarction in the emergency setting. Circulation journal: official journal of the Japanese Circulation Society.

[39] He Y, Ma C, Xing J, Wang S, Ji C, Han Y, Zhang J (2019). Serum amyloid a protein as a potential biomarker in predicting acute onset and association with in-hospital death in acute aortic dissection. BMC Cardiovasc Disord.

[40] Nazerian P, Morello F, Vanni S, Bono A, Castelli M, Forno D, Gigli C, Soardo F, Carbone F, Lupia E (2014). Combined use of aortic dissection detection risk score and D-dimer in the diagnostic workup of suspected acute aortic dissection. Int J Cardiol.

[41] Ohlmann P, Faure A, Morel O, Petit H, Kabbaj H, Meyer N, Cheneau E, Jesel L, Epailly E, Desprez D (2006). Diagnostic and prognostic value of circulating D-Dimers in patients with acute aortic dissection. Crit Care Med.

[42] Okazaki T, Yamamoto Y, Yoda K, Nagahiro S (2014). The ratio of D-dimer to brain natriuretic peptide may help to differentiate between cerebral infarction with and without acute aortic dissection. J Neurol Sci.

[43] Sbarouni E, Georgiadou P, Marathias A, Geroulanos S, Kremastinos DT (2007). D-dimer and BNP levels in acute aortic dissection. Int J Cardiol.

[44] Shao N, Xia S, Wang J, Zhou X, Huang Z, Zhu W, Chen Y (2014). The role of D-dimers in the diagnosis of acute aortic dissection. Mol Biol Rep.

[45] Weber T, Högler S, Auer J, Berent R, Lassnig E, Kvas E, Eber B (2003). D-dimer in acute aortic dissection. Chest.

[46] Tian C, Tang X, Zhu X, Zhou Q, Guo Y, Zhao R, Wang D, Gong B (2019). Expression profiles of circRNAs and the potential diagnostic value of serum circMARK3 in human acute Stanford type A aortic dissection. PLoS ONE.

[47] Senturk T, Antal A, Gunel T (2019). Potential function of microRNAs in thoracic aortic aneurysm and thoracic aortic dissection pathogenesis. Mol Med Rep.

[48] Xu Z, Wang Q, Pan J, Sheng X, Hou D, Chong H, Wei Z, Zheng S, Xue Y, Zhou Q (2017). Characterization of serum miRNAs as molecular biomarkers for acute Stanford type A aortic dissection diagnosis. Sci Rep.

[49] Jones CM, Athanasiou T (2005). Summary receiver operating characteristic curve analysis techniques in the evaluation of diagnostic tests. Ann Thorac Surg.

[50] Youden WJ (1950). Index for rating diagnostic tests. Cancer.

[51] Lai CY, Tian L, Schisterman EF (2012). Exact confidence interval estimation for the Youden index and its corresponding optimal cut-point. Comput Stat Data Anal.

[52] Hoeprich PD, Doolittle RF (1983). Dimeric half-molecules of human fibrinogen are joined through disulfide bonds in an antiparallel orientation. Biochemistry.

[53] Favresse J, Lippi G, Roy PM, Chatelain B, Jacqmin H, Ten Cate H, Mullier F (2018). D-dimer: Preanalytical, analytical, postanalytical variables, and clinical applications. Crit Rev Clin Lab Sci.

[54] Mosesson MW, Siebenlist KR, Hainfeld JF, Wall JS (1995). The covalent structure of factor XIIIa crosslinked fibrinogen fibrils. J Struct Biol.

[55] Longstaff C, Kolev K (2015). Basic mechanisms and regulation of fibrinolysis. J Thromb haemostasis: JTH.

[56] Hager K, Platt D (1995). Fibrin degeneration product concentrations (D-dimers) in the course of ageing. Gerontology.

[57] Huang X, Yue Z, Wu J, Chen J, Wang S, Wu J, Ren L, Zhang A, Deng P, Wang K (2018). MicroRNA-21 knockout exacerbates Angiotensin II-Induced thoracic aortic aneurysm and dissection in mice with abnormal transforming growth Factor-β-SMAD3 signaling. Arterioscler Thromb Vasc Biol.

[58] Sun J, Chen G, Jing Y, He X, Dong J, Zheng J, Zou M, Li H, Wang S, Sun Y (2018). LncRNA expression Profile of Human thoracic aortic dissection by high-throughput sequencing. Cell Physiol biochemistry: Int J experimental Cell Physiol Biochem Pharmacol.

[59] Xiao Y, Sun Y, Ma X, Wang C, Zhang L, Wang J, Wang G, Li Z, Tian W, Zhao Z (2020). MicroRNA-22 inhibits the apoptosis of vascular smooth muscle cell by targeting p38MAPKα in vascular remodeling of aortic dissection. Mol therapy Nucleic acids.

[60] Yang P, Wu P, Liu X, Feng J, Zheng S, Wang Y, Fan Z (2020). MiR-26b suppresses the development of Stanford Type A aortic dissection by regulating HMGA2 and TGF-β/Smad3 signaling pathway. Annals of thoracic and cardiovascular surgery: official journal of the Association of Thoracic and Cardiovascular Surgeons of Asia.

[61] Tieu BC, Lee C, Sun H, Lejeune W, Recinos A, Ju X, Spratt H, Guo DC, Milewicz D, Tilton RG (2009). An adventitial IL-6/MCP1 amplification loop accelerates macrophage-mediated vascular inflammation leading to aortic dissection in mice. J Clin Investig.

[62] del Porto F, Proietta M, Tritapepe L, Miraldi F, Koverech A, Cardelli P, Tabacco F, de Santis V, Vecchione A, Mitterhofer AP (2010). Inflammation and immune response in acute aortic dissection. Ann Med.

[63] Park SL, Hwang B, Lee SY, Kim WT, Choi YH, Chang YC, Kim WJ, Moon SK (2015). p21WAF1 is required for Interleukin-16-Induced Migration and Invasion of Vascular smooth muscle cells via the p38MAPK/Sp-1/MMP-9 pathway. PLoS ONE.

