# Supplementary material for: Diagnostic biomarkers and aortic dissection: a systematic review and meta-analysis
Source: BMC Cardiovasc Disord. 2023 Oct 10;23:497. doi: 10.1186/s12872-023-03448-9 (PMC10563263; doi:10.1186/s12872-023-03448-9)
Supplement: Supplementary file 1 — Supplementary Material 1 [file 12872_2023_3448_MOESM1_ESM.docx]

**Diagnostic Biomarkers and Aortic Dissection: A Systematic Review and meta-analysis**

Hongjian Chen ^1, *^, Yunjie Li,^2, *^, Zheqian Li ^3, *^, Yanli Shi,^3, #^, Haobo Zhu ^4, #^

**Supplement Legend**

**Supplement figure 1 Assessment of methodological quality of the studies included in the review by quality assessment of diagnostic accuracy studies 2 (QUADAS-2).**

**Supplement figure 2 Meta-regression analysis for D-dimer.**

**Supplement figure 3 Diagnostic accuracy for D-dimer of research before year 2017 (A) Diagnostic sensitivity and specificity (B) Diagnostic accuracy (C) Receiver operating characteristic curve (ROC) (D) Publication bias.**

**Supplement figure 4 Diagnostic accuracy for D-dimer of research after year 2017 (A) Diagnostic sensitivity and specificity (B) Diagnostic accuracy (C) Receiver operating characteristic curve (ROC) (D) Publication bias.**

**Supplement figure 5 Diagnostic accuracy for D-dimer of research sample size greater than 100 (A) Diagnostic sensitivity and specificity (B) Diagnostic accuracy (C) Receiver operating characteristic curve (ROC) (D) Publication bias.**

**Supplement figure 6 Diagnostic accuracy for D-dimer of research sample size less than 100 (A) Diagnostic sensitivity and specificity (B) Diagnostic accuracy (C) Receiver operating characteristic curve (ROC) (D) Publication bias.**

**Supplement figure 7 Diagnostic accuracy for D-dimer of research in Asian (A) Diagnostic sensitivity and specificity (B) Diagnostic accuracy (C) Receiver operating characteristic curve (ROC) (D) Publication bias.**

**Supplement figure 8 Diagnostic accuracy for D-dimer of research in non-Asian (A) Diagnostic sensitivity and specificity (B) Diagnostic accuracy (C) Receiver operating characteristic curve (ROC) (D) Publication bias.**

**Supplementary Table 1 Detail of search strategies and results for different databases.**

**Supplementary Table 2 Characteristics of studies about other biomarkers included in the analysis.**

**Supplementary Table 3 The role of 9 biomarkers in the pathological development of aortic dissection.**

**Supplementary Table 4 Function of disordered representative ncRNA in aortic dissection.**

**Supplementary Table 5 Function of disordered ncRNA in aortic dissection.**

**Supplementary Table 6 Summary of systematic reviews evaluating the diagnostic accuracy of D-dimer for aortic dissection**.

**Supplementary Table 1 Detail of search strategies and results for different databases.**

|  |  | **Search Strategies** | **Result** |
| --- | --- | --- | --- |
| **Pubmed** | **1** | (((((((("Plasma"[Mesh]) OR Plasmas) OR Blood Plasma) OR Blood Plasmas) OR Plasma, Blood) OR Plasmas, Blood) OR (((("Serum"[Mesh]) OR (Serums)) OR (Blood Serum)) OR (Serum, Blood))) AND (((((((("Aneurysm, Dissecting"[Mesh]) OR (Dissection, Blood Vessel)) OR (Blood Vessel Dissection)) OR (Aortic Dissection)) OR (Aortic Dissections)) OR (Dissection, Aortic)) OR (Dissections, Aortic)) AND ((((("Diagnosis"[Mesh]) OR (Diagnoses)) OR (Diagnose)) OR (Diagnoses and Examinations)) OR (Examinations and Diagnoses)))) OR ( "Aneurysm, Dissecting/blood"[Mesh] OR "Aneurysm, Dissecting/diagnosis"[Mesh] ) NOT ("Review" [Publication Type]) NOT ("Comment" [Publication Type]) | 10575 |
| **Web of Science** | **1** | TS=("Plasma" OR "Plasmas" OR "Blood Plasma" OR "Blood Plasmas" OR "Plasma, Blood" OR "Plasmas, Blood" OR "Serum"  OR "Serums" OR "Blood Serum" OR "Serum, Blood") | 3478627 |
|  | 2 | TS=("Aneurysm, Dissecting" OR "Dissection, Blood Vessel" OR  "Blood VesselDissection" OR "Aortic Dissection" OR "Aortic  Dissections" OR "Dissection, Aortic" OR "Dissections, Aortic") | 30755 |
|  | 3 | TS=(diagno*) | 8647738 |
|  |  | #3 AND #2 AND #1 | 484 |
| **EMbase** | **1** | ((((((((Plasma) OR Plasmas) OR Blood Plasma) OR Blood Plasmas) OR Plasma, Blood) OR Plasmas, Blood) OR ((((Serum) OR (Serums)) OR (Blood Serum)) OR (Serum, Blood))) AND ((((((((Aneurysm, Dissecting) OR (Dissection, Blood Vessel)) OR (Blood Vessel Dissection)) OR (Aortic Dissection)) OR (Aortic Dissections)) OR (Dissection, Aortic)) OR (Dissections, Aortic)) AND (((((Diagnosis) OR (Diagnoses)) OR (Diagnose)) OR (Diagnoses and Examinations)) OR (Examinations and Diagnoses)))) | 961 |
| **Cochrane Library** | **1** | MeSH descriptor: [Plasma] explode all trees | 1113 |
|  | 2 | MeSH descriptor: [Aneurysm, Dissecting] explode all trees | 115 |
|  | 3 | (Dissection, Blood Vessel OR Blood Vessel Dissection OR Aortic Dissection OR Aortic Dissections OR Dissection, Aortic OR Dissections, Aortic) | 891 |
|  | 4 | MeSH descriptor: [Diagnosis] explode all trees | 342030 |
|  | 5 | (Diagnoses OR Diagnose OR Diagnoses and Examinations OR Examinations and Diagnoses) | 161898 |
|  | 6 | (Plasmas OR Blood Plasma OR Blood Plasmas OR Plasma, Blood OR Plasmas, Blood OR Serum OR Serums OR Blood Serum OR Serum, Blood) | 172780 |
|  | 7 | MeSH descriptor: [Serum] explode all trees | 883 |
|  |  | ((#1 OR #7 OR #6) AND (#2 OR #3) AND (#5 AND #4)) | 6 |

**Supplementary Table 2 Characteristics of studies about other biomarkers included in the analysis.**

| No | Author | Year | Sample | Type of disease | Biomarker | AUC | Sen | Spe | Youden index | No. Case | No. Control | cut-off value | Reference |
| --- | --- | --- | --- | --- | --- | --- | --- | --- | --- | --- | --- | --- | --- |
| 1 | Fan | 2020 | blood | TAAD | OPN | 0.9898 | 0.9200 | 0.9900 | 0.9100 | 50 | 25 | NP | [1] |
| 2 | Li | 2017 | serum | AAAD | ADAMTS4 | 0.9893 | 0.9459 | 0.9706 | 0.9165 | 74 | 70 | NP | [2] |
| 3 | Xiao | 2020 | blood | AAD | MHR | 0.9790 | 0.9804 | 0.9364 | 0.9168 | 128 | 110 | NP | [3] |
| 4 | Li | 2017 | serum | AAAD | ADAMTS1 | 0.9710 | 0.8784 | 0.9706 | 0.8490 | 74 | 70 | NP | [2] |
| 5 | Wang | 2018 | plasma | AAD | sST2 | 0.9700 | 0.9500 | 0.9800 | 0.9300 | 144 | 219 | NP | [4] |
| 6 | König | 2021 | plasma | AAAD | Aggrecan (ACAN) | 0.9470 | 0.9700 | 0.8100 | 0.7800 | 33 | 66 | 14.3 ng/mL | [5] |
| 7 | He | 2019 | serum | AAD | Serum amyloid A (SAA) | 0.9420 | 0.9080 | 0.9370 | 0.8450 | 63 | 87 | 0.427mg/L | [6] |
| 8 | He | 2019 | serum | TAAD | Serum amyloid A (SAA) | 0.9390 | 0.9190 | 0.9370 | 0.8560 | 31 | 87 | 0.427mg/L | [6] |
| 9 | He | 2019 | serum | TBAD | Serum amyloid A (SAA) | 0.9370 | 0.8810 | 0.9370 | 0.8180 | 50 | 87 | 0.462mg/L | [6] |
| 10 | Ma | 2021 | plasma | BAAD | Ceruloplasmin (CP) | 0.9340 | 0.9530 | 0.8820 | 0.8350 | 40 | 85 | 32.50 mg/dL | [7] |
| 11 | Ma | 2021 | plasma | AAD | Ceruloplasmin (CP) | 0.9290 | 0.9060 | 0.9290 | 0.8350 | 102 | 85 | 36.82 mg/dL | [7] |
| 12 | Ma | 2021 | plasma | AAAD | Ceruloplasmin (CP) | 0.9220 | 0.9000 | 0.9290 | 0.8290 | 45 | 85 | 37.11 mg/dL | [7] |
| 13 | Peng | 2015 | serum | AAD | PC1 | 0.9000 | 0.8571 | 0.7561 | 0.6132 | 35 | 52 | >357.33(pg/ml) | [8] |
| 14 | Xiao | 2016 | serum | AAD | Lumican | 0.8950 | 0.7333 | 0.9833 | 0.7166 | 60 | 60 | 2.19 ng/mL | [9] |
| 15 | Han | 2021 | Venous  blood | AAD | S100A1 | 0.8900 | 0.8440 | 0.8550 | 0.6990 | 77 | 76 | 1.10 ng/mL | [10] |
| 16 | Han | 2021 | Venous  blood | AAD | hs-CRP | 0.8800 | NP | NP | NP | 77 | 76 | NP | [10] |
| 17 | Nagaoka | 2010 | plasma | AAD | fibrinogen/fibrin degradation products (FDP) | 0.8700 | 0.9800 | 0.5400 | 0.5200 | 50 | 57 | 2.05 μg/mL | [11] |
| 18 | Cakir | 2021 | blood | AAD | SCUBE-1 | 0.8630 | 0.9500 | 0.7600 | 0.7100 | 20 | 20 | NP | [12] |
| 19 | Han | 2021 | Venous  blood | AAD | cTnT | 0.8500 | NP | NP | NP | 77 | 76 | NP | [10] |
| 20 | Fan | 2020 | blood | TAAD | MCP-2 | 0.8364 | NP | NP | NP | 50 | 25 | NP | [1] |
| 21 | Forrer | 2021 | plasma | AAD | IL-10 | 0.8300 | 0.5500 | 0.9800 | 0.5300 | 34 | 150 | 20 ng/L | [13] |
| 22 | Peng | 2015 | serum | AAD | sELAF | 0.8200 | 0.8286 | 0.6829 | 0.5115 | 35 | 52 | >97.07(ng/ml) | [8] |
| 23 | Li | 2018 | serum | AAD | MMP9 | 0.8100 | 0.6820 | 0.8410 | 0.5230 | 88 | 88 | 379.47ng/ml | [14] |
| 24 | Peng | 2015 | serum | AAD | smMHC | 0.8100 | 0.6857 | 0.9024 | 0.5881 | 35 | 52 | >2.11(ng/ml) | [8] |
| 25 | Yang | 2020 | blood | TAD | hs-CRP | 0.8070 | 0.6790 | 0.8920 | 0.5710 | 78 | 72 | NP | [15] |
| 26 | Fan | 2020 | blood | TAAD | ENA-78 | 0.8068 | NP | NP | NP | 50 | 25 | NP | [1] |
| 27 | Fan | 2020 | blood | TAAD | MCP-1 | 0.8020 | NP | NP | NP | 50 | 25 | NP | [1] |
| 28 | Li | 2018 | serum | AAD | TLR4 | 0.7990 | 0.7500 | 0.7050 | 0.4550 | 88 | 88 | 7.83ng/ml | [14] |
| 29 | Forrer | 2021 | plasma | AAD | PAI1 | 0.7800 | 0.4800 | 0.9700 | 0.4500 | 34 | 150 | 58 µg/L | [13] |
| 30 | Fan | 2020 | blood | TAAD | IL-16 | 0.7714 | NP | NP | NP | 50 | 25 | NP | [1] |
| 31 | Forrer | 2021 | plasma | AAD | IL-6 | 0.7500 | NP | NP | NP | 34 | 150 | NP | [13] |
| 32 | Forrer | 2021 | plasma | AAD | IGFBP1 | 0.7500 | NP | NP | NP | 34 | 150 | NP | [13] |
| 33 | Giachino | 2013 | plasma | AAD | MMP8 | 0.7500 | 1.0000 | 0.0950 | 0.0950 | 52 | 74 | 3.6 ng/ml | [16] |
| 34 | Yang | 2020 | blood | TAD | ANGPTL8 | 0.7460 | 0.7950 | 0.6210 | 0.4160 | 78 | 72 | NP | [15] |
| 35 | Forrer | 2021 | plasma | AAD | IL-1ra | 0.7100 | NP | NP | NP | 34 | 150 | NP | [13] |
| 36 | Giachino | 2013 | plasma | AAD | MMP9 | 0.7000 | 0.9620 | 0.1620 | 0.1240 | 52 | 74 | 20.0 ng/ml | [16] |
| 37 | Toru | 2008 | blood | AAAD | Basic calponinb | 0.6700 | NP | NP | NP | 16 | 52 | NP | [17] |
| 38 | Toru | 2008 | blood | AAAD | Acidic calponina | 0.6600 | NP | NP | NP | 59 | 158 | NP | [17] |
| 39 | Toru | 2008 | blood | AAAD | Basic calponinb | 0.6500 | NP | NP | NP | 59 | 158 | NP | [17] |
| 40 | Toru | 2008 | blood | BAAD | Acidic calponina | 0.6500 | NP | NP | NP | 15 | 158 | NP | [17] |
| 41 | Fan | 2020 | blood | TAAD | MIP-1β | 0.6404 | NP | NP | NP | 50 | 25 | NP | [1] |
| 42 | Toru | 2008 | blood | AAAD | Acidic calponina | 0.6300 | NP | NP | NP | 16 | 52 | NP | [17] |
| 43 | Toru | 2008 | blood | BAAD | Acidic calponina | 0.6300 | NP | NP | NP | 2 | 52 | NP | [17] |
| 44 | Peng | 2015 | serum | AAD | α-SMA | 0.6200 | 0.5429 | 0.9024 | 0.4453 | 35 | 52 | >49.62(ng/ml) | [8] |
| 45 | Toru | 2008 | blood | BAAD | Basic calponinb | 0.5900 | NP | NP | NP | 15 | 158 | NP | [17] |
| 46 | Xiao | 2016 | serum | AAD | CRP | 0.5860 | 0.3833 | 0.8833 | 0.2666 | 60 | 60 | 36.8 mg/L | [9] |
| 47 | Toru | 2008 | blood | BAAD | Basic calponinb | 0.5800 | NP | NP | NP | 2 | 52 | NP | [17] |
| 48 | Xiao | 2016 | serum | AAD | TSP-1 | 0.5510 | 0.9833 | 0.4500 | 0.4333 | 60 | 60 | 2564.5 ng/mL | [9] |
| 49 | Forrer | 2021 | plasma | AAD | hs-TnT | 0.5100 | NP | NP | NP | 34 | 150 | NP | [13] |
| 50 | Toru | 2008 | blood | BAAD | Neutral calponin | 0.5000 | NP | NP | NP | 2 | 52 | NP | [17] |
| 51 | Wang | 2018 | plasma | AAD | cTnI | 0.5000 | 0.4400 | 0.5600 | 0.0000 | 113 | 216 | NP | [4] |
| 52 | Toru | 2008 | blood | BAAD | Neutral calponin | 0.4900 | NP | NP | NP | 15 | 158 | NP | [17] |
| 53 | Toru | 2008 | blood | AAAD | Neutral calponin | 0.4200 | NP | NP | NP | 16 | 52 | NP | [17] |
| 54 | Toru | 2008 | blood | AAAD | Neutral calponin | 0.3900 | NP | NP | NP | 59 | 158 | NP | [17] |
| 55 | Suzuki | 2000 | blood | AAD | Smooth-Muscle Myosin Heavy-Chain Protein | NP | 0.9800 | 0.9600 | 0.9400 | 95 | 131 | 2.5 µg/L | [18] |

NP, not report.

**Supplementary Table 3 The role of 9 biomarkers in the pathological development of aortic dissection.**

| **Biomarkers** | **Function** |
| --- | --- |
| Osteopontin (OPN) | OPN is related to the phenotypic transition of VSMCs in AD, is highly expressed in synthetic phenotype vascular smooth muscle cells, and can promote the proliferation, migration and anti-apoptosis of vascular smooth muscle cells[19-24]; |
| ADAMTS1 | ADAMTS1 degrades extracellular matrix proteins and damages the aortic wall; ADAMTS1 promotes the migration of centriocytes, the migration and infiltration of macrophages; ADAMTS1 affects the proliferation, apoptosis and migration of VSMCs[25-33]; |
| ADAMTS4 | ADAMTS4 degrades extracellular matrix proteins and damages the aortic wall; ADAMTS4 promotes migration and infiltration of macrophages;  ADAMTS4 affects the apoptosis of VSMCs[34-39]; |
| Soluble ST2 (sST2) | sST2 promotes ECM remodeling and turnover in VSMCs; IL-33/ST2L has a direct pro-inflammatory effect on endothelial cells and promotes vascular permeability[40-45]; |
| Aggrecan (ACAN) | Massive accumulation of Aggrecan disrupts ECM osmotic pressure in VSMCs  Massive accumulation of Aggrecan may affect the viability of VSMCs[46]; |
| Serum amyloid A (SAA) | SAA promotes phenotypic switch and migration of VSMCs;  SAA induces inflammation in VSMCs[47-51]; |
| Ceruloplasmin (CP) | CP may be elevated in response to elevated ROS in AD[52-59]; |
| Polycystin 1 (PC1) | PC1 regulates the development of VSMCs; Downregulation of PC1 promotes phenotypic switch, proliferation and ECM dysfunction in VSMCs[60-63]; |
| Monocyte to High-density Lipoprotein Ratio (MHR) | MHR can be used as a biomarker for inflammation-related diseases[64-69]; |

**Supplementary Table 4 Function of disordered representative ncRNA in aortic dissection.**

| ncRNA | Sample | Expression | Cell | Target | Function of disordered ncRNA in cell | References |
| --- | --- | --- | --- | --- | --- | --- |
| miR‐21 | Aorta | Up | VSMC | SMAD7 | promote phenotypic transition | [70] |
| miR‐134‐5p | Aorta | Down | VSMC | STAT5B and ITGB1 | inhibit phenotypic transition promote proliferation and migration | [71] |
| miR‐145 | Aorta | Down | VSMC | CTGF | inhibit proliferation and promote apoptosis | [72] |
| miR‐320 | Peripheral blood | Down | Monocytes  Macrophage | MMP2/9 | promote degradation of the ECM | [73] |
| miR‐320d | Aorta | Down | VSMC | TRIAP1 and NET1 | inhibit apoptosis | [74] |
| miR‐582 | Aorta | Down | VSMC | TRIAP1 and NET1 | inhibit apoptosis | [74] |
| miR‐144‐3p | Aorta | Up | VSMC | TE | increase the incidence and severity of AD | [75] |
| miR‐146a‐5p | Aorta and plasma | Up | VSMC | SMAD4 | promote proliferation and migration | [76] |
| miR‐143/miR‐145 | Aorta | Down | VSMC | TGF‐β1 | promote phenotypic transition | [77] |
| miR-193a-3p | Aorta | Up | VSMC | ACTG2 | promote phenotypic transition promote proliferation and migration | [78] |
| miR-22-3p | Aorta | Down | VSMC | p38MAPKα | promote apoptosis | [79] |
| miR-107-5p | Aorta | Up | VSMC | ITM2C | promote proliferation and inhibit apoptosis | [80] |
| miR-26b | Aorta | Down | VSMC | HMGA2 | promote apoptosis and inhibit proliferation | [81] |
| miR-27a | Aorta | Down | endothelial cells (ECs) | FADD | promote apoptosis and migration | [82] |
| miR-124 | Aorta | Down | VSMC | Sp1 | promote phenotypic transition promote proliferation | [83] |
| CDKN2B‐AS1 | Aorta | Up | VSMC | miR-320d | inhibit proliferation promote apoptosis | [84] |
| lncRNA‐XIST | Aorta | Up | VSMC | miR-17/PTEN | inhibit proliferation promote apoptosis | [85] |
| lncRNA‐XIST | Aorta | Up | VSMC | has-miR-17-5p/p21 | Inhibit proliferation | [86] |
| PTENP1 | Aorta | Up | VSMC | miR‐21 | Promote apoptosis | [87] |
| linc01278 | Aorta | Down | VSMC | miR-500b-5p/ACTG2 | inhibit phenotypic transition promote proliferation and migration | [88] |
| lncRNA OIP5-AS1 | Aorta | Up | VSMC | miR-143-3p/TUB | inhibit proliferation and migration promote apoptosis | [89] |
| lncRNA H19 | Aorta | Up | VSMC | miR-193b-3p | inhibit phenotypic transition promote proliferation and migration | [90] |
| lncRNA PVT1 | Aorta | Up | VSMC | miR-27b-3p | promote phenotypic transition promote proliferation and migration | [91] |

**Supplementary Table 5 Function of disordered ncRNA in aortic dissection.**

| ncRNA | Sample | Expression | References |
| --- | --- | --- | --- |
| lncP2RX7 | Aorta | Up | [92] |
| HIF1A‐AS2 | Aorta | Up | [92] |
| AX746823 | Aorta | Up | [92] |
| RP11‐69I8.3 | Aorta | Up | [92] |
| RP11‐536K7.5 | Aorta | Up | [92] |
| ENSG00000269936 | Aorta | Up | [86] |
| lncRNA‐1421 | Aorta | Down | [86] |
| ENSG00000248508 | Aorta | Up | [86] |
| ENSG00000226530 | Aorta | Up | [86] |
| EG00000259719 | Aorta | Up | [86] |
| circMARK3 | Aorta and serum | Up | [93] |
| hsa_circRNA_101238 | Aorta | Up | [94] |
| hsa_circRNA_104634 | Aorta | Up | [94] |
| hsa_circRNA_002271 | Aorta | Up | [94] |
| hsa_circRNA_102771 | Aorta | Up | [94] |
| hsa_circRNA_104349 | Aorta | Up | [94] |
| hsa_circRNA_102683 | Aorta | Down | [94] |
| hsa_circRNA_005525 | Aorta | Down | [94] |
| hsa_circRNA_103458 | Aorta | Down | [94] |

**Supplementary Table 6 Summary of systematic reviews evaluating the diagnostic accuracy of D-dimer for aortic dissection**.

| Author | Year | Cut-off value (ng/mL) | AUC | Sen | Spe | DOR |  | References |
| --- | --- | --- | --- | --- | --- | --- | --- | --- |
| Sodeck | 2007 | 100–900 | 0.94 | 0.97 | 0.59 | 21.27 |  | [95] |
| Marill | 2008 | 500 | NA | 0.94 | 0.95 | NP |  | [96] |
| Shimon | 2011 | 500 | NA | 0.97 | 0.56 | NP |  | [97] |
| Cui | 2015 | 170–5000 | 0.92 | 0.945 | 0.691 | NP |  | [98] |
| Asha | 2015 | 400–500 | NA | 0.98 | 0.419 | NP |  | [99] |
| Watanabe | 2016 | 246–8700 | 0.946 | NA | NA | 28.5 |  | [100] |
| Watanabe | 2016 | 500 | 0.95 | 0.952 | 0.604 | 30.7 |  | [100] |

AUC, area under the curve of receiver operator characteristics. Sen, sensitivity. Spe, specificity. DOR, diagnostic odds ratio. NP, not report.

**Supplement figure 1 Assessment of methodological quality of the studies included in the review by quality assessment of diagnostic accuracy studies 2 (QUADAS-2).**


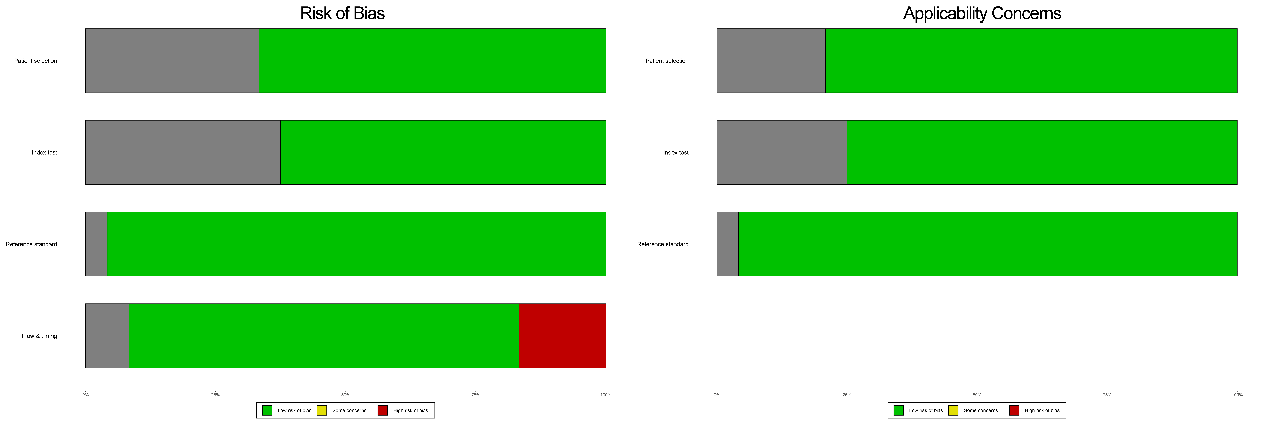


**Supplement figure 2 Meta-regression analysis for D-dimer.**

**
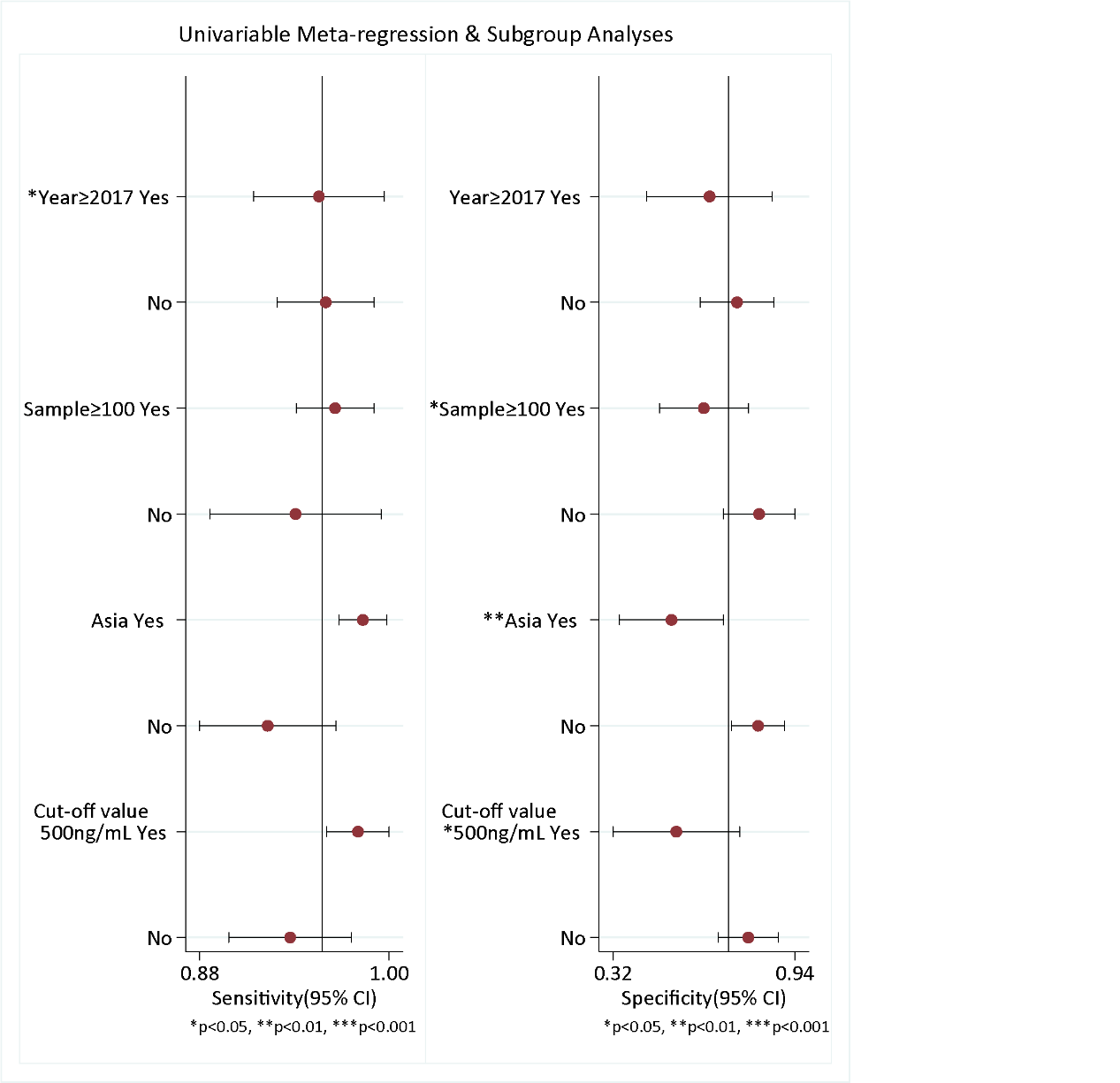
**

**Supplement figure 3 Diagnostic accuracy for D-dimer of research before year 2017**

**
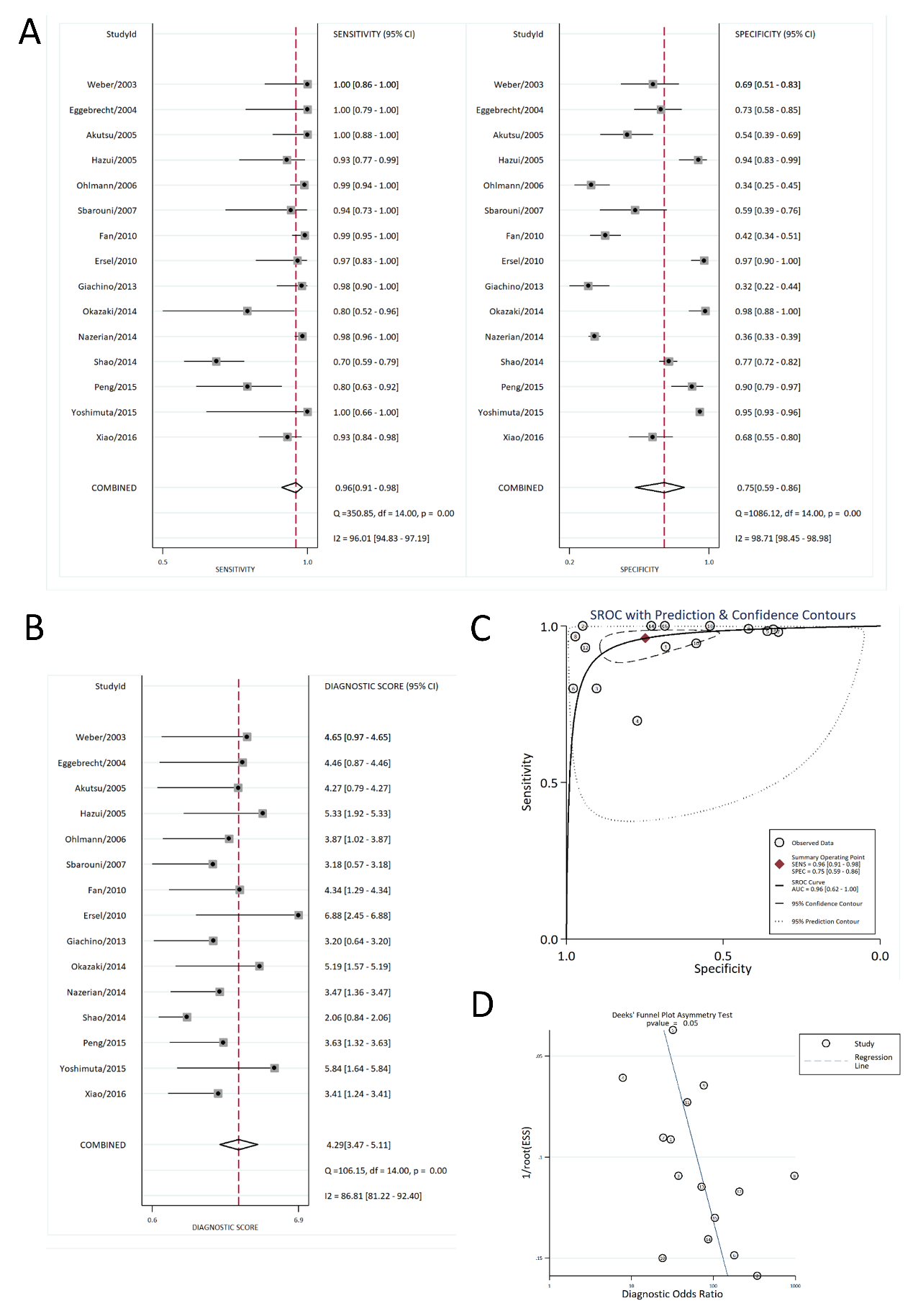
**

**(A) Diagnostic sensitivity and specificity (B) Diagnostic accuracy (C) Receiver operating characteristic curve (ROC) (D) Publication bias.**

**Supplement figure 4 Diagnostic accuracy for D-dimer of research after year 2017**

**
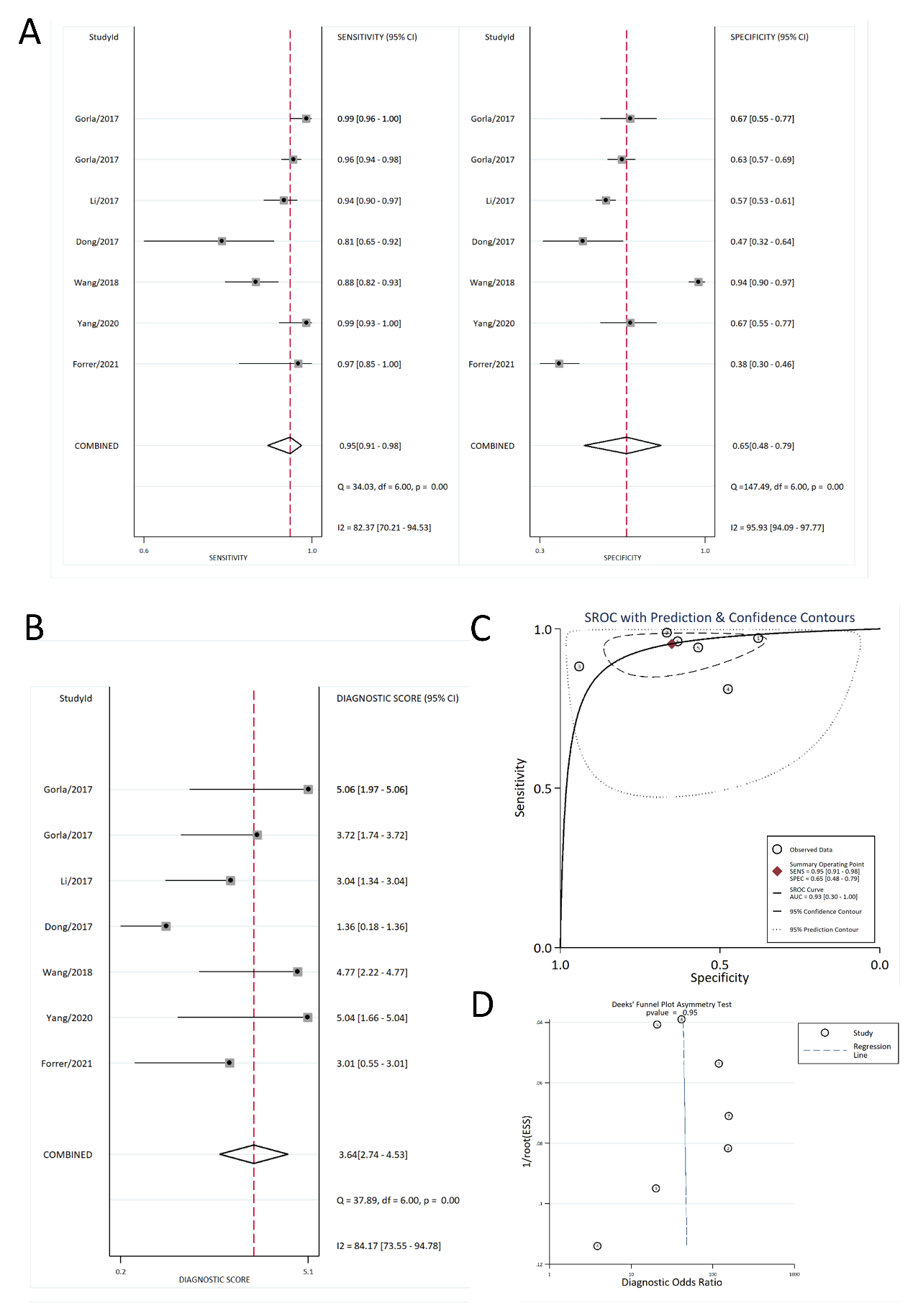
(A) Diagnostic sensitivity and specificity (B) Diagnostic accuracy (C) Receiver operating characteristic curve (ROC) (D) Publication bias.**

**Supplement figure 5 Diagnostic accuracy for D-dimer of research sample size greater than 100**

**
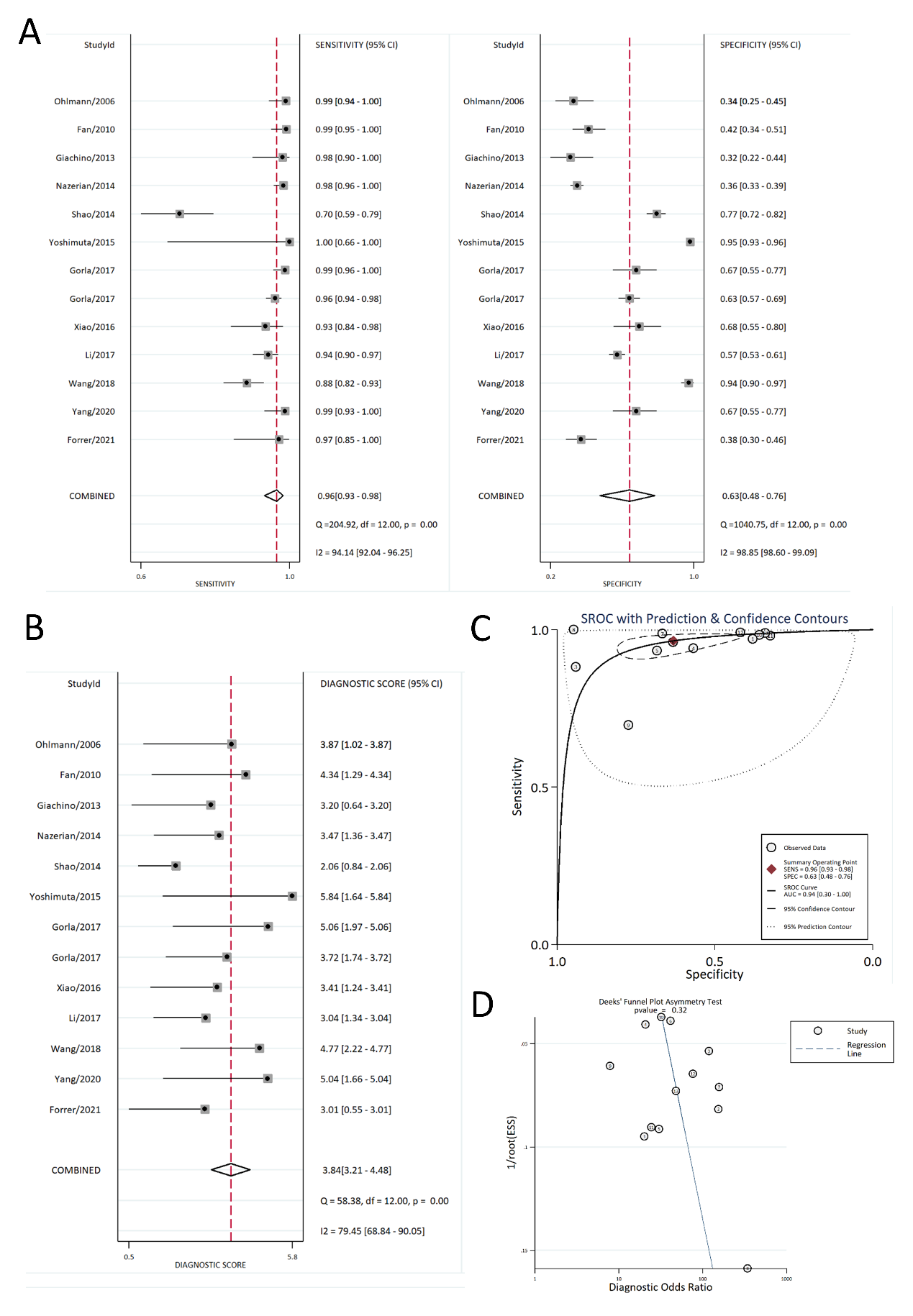
(A) Diagnostic sensitivity and specificity (B) Diagnostic accuracy (C) Receiver operating characteristic curve (ROC) (D) Publication bias.**

**Supplement figure 6 Diagnostic accuracy for D-dimer of research sample size less than 100**

**
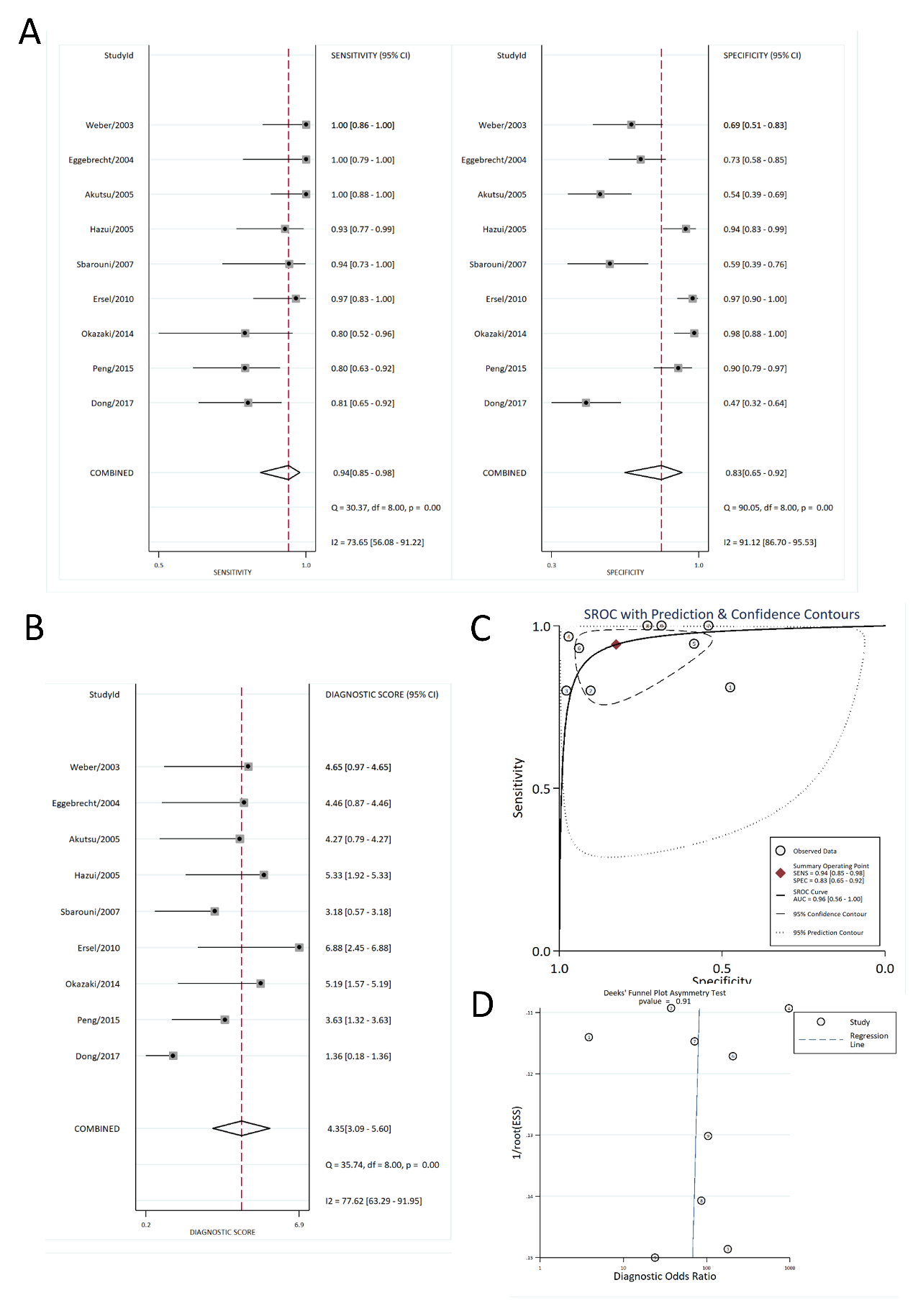
**

**(A) Diagnostic sensitivity and specificity (B) Diagnostic accuracy (C) Receiver operating characteristic curve (ROC) (D) Publication bias.**

**Supplement figure 7 Diagnostic accuracy for D-dimer of research in Asian**

**
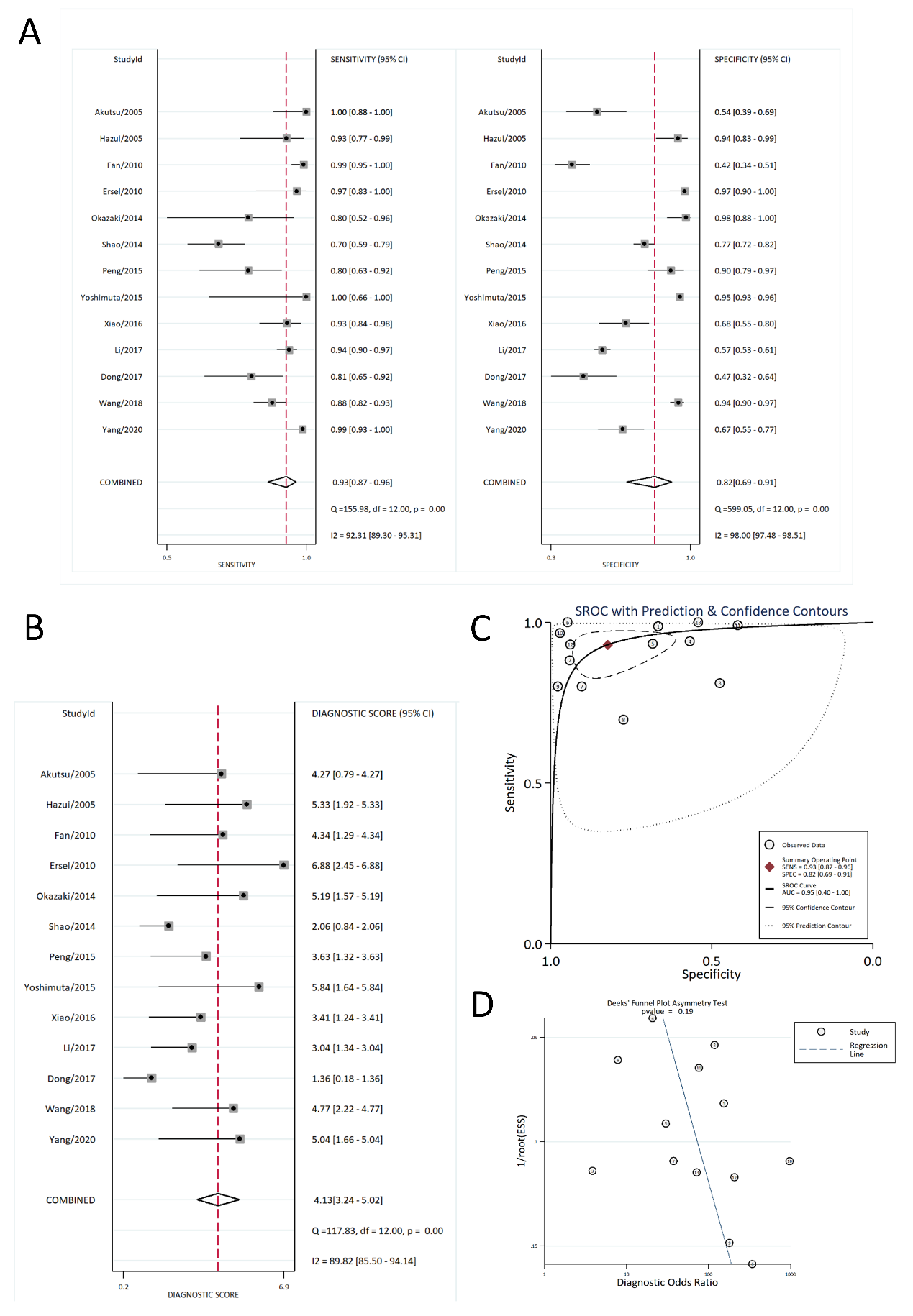
**

**(A) Diagnostic sensitivity and specificity (B) Diagnostic accuracy (C) Receiver operating characteristic curve (ROC) (D) Publication bias.**

**Supplement figure 8 Diagnostic accuracy for D-dimer of research in non-Asian**

**
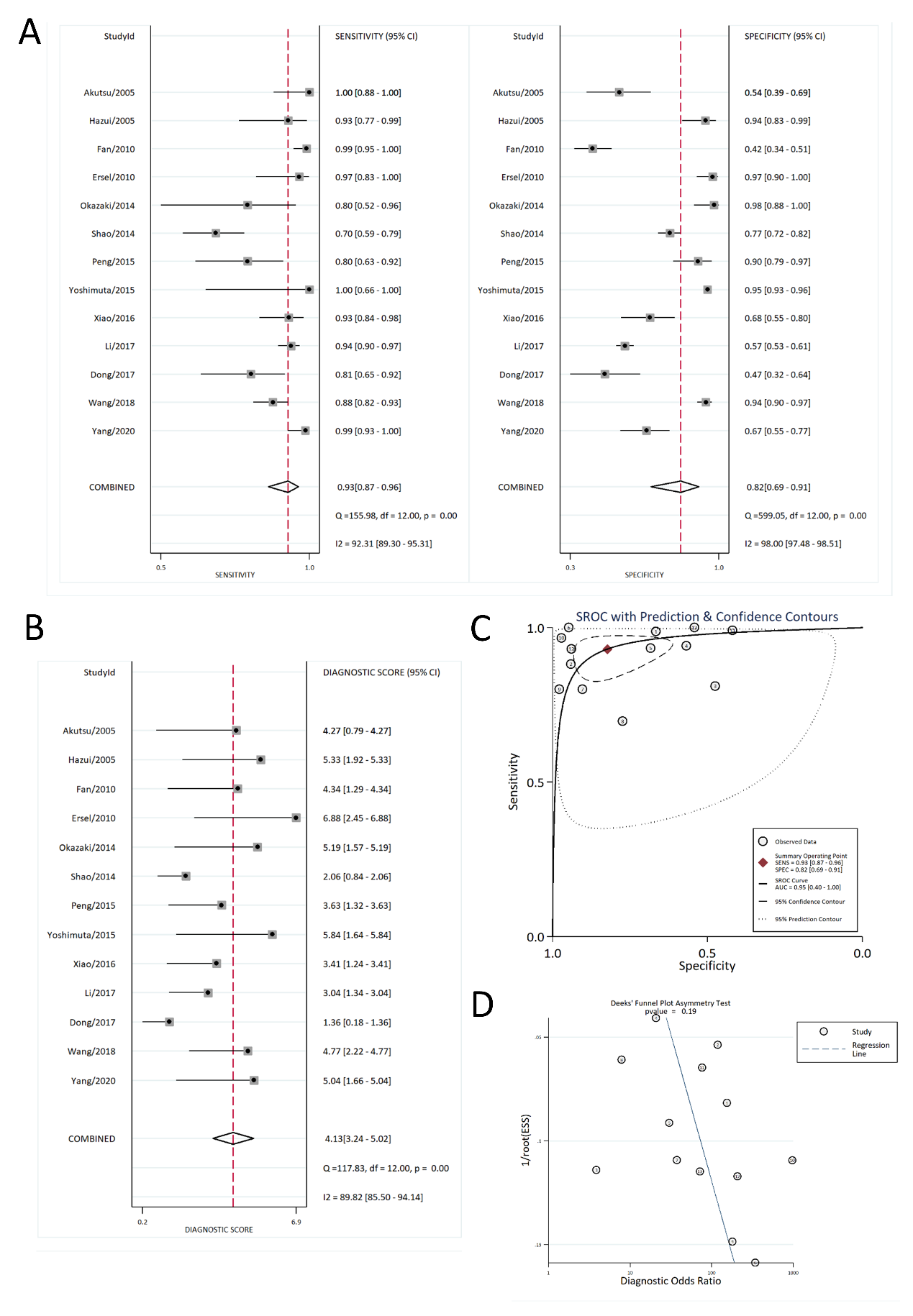
**

**(A) Diagnostic sensitivity and specificity (B) Diagnostic accuracy (C) Receiver operating characteristic curve (ROC) (D) Publication bias.**

**Reference**

[1] Fan F, Zhou Q, Pan J, Wang Q, Cao H, Xue Y, et al. Preliminary observation of chemokine expression in patients with Stanford type A aortic dissection. Cytokine. 2020;127:154920.

[2] Li K, Wang ZW, Hu Z, Ren Z, Hu X, Li L, et al. Assessing Serum Levels of ADAMTS1 and ADAMTS4 as New Biomarkers for Patients with Type A Acute Aortic Dissection. Medical science monitor : international medical journal of experimental and clinical research. 2017;23:3913-22.

[3] Du X, Zhang S, Xu J, Xiang Q, Tian F, Li X, et al. Diagnostic value of monocyte to high-density lipoprotein ratio in acute aortic dissection in a Chinese han population. Expert review of molecular diagnostics. 2020;20:1243-52.

[4] Wang Y, Tan X, Gao H, Yuan H, Hu R, Jia L, et al. Magnitude of Soluble ST2 as a Novel Biomarker for Acute Aortic Dissection. Circulation. 2018;137:259-69.

[5] König KC, Lahm H, Dreßen M, Doppler SA, Eichhorn S, Beck N, et al. Aggrecan: a new biomarker for acute type A aortic dissection. Scientific reports. 2021;11:10371.

[6] He Y, Ma C, Xing J, Wang S, Ji C, Han Y, et al. Serum amyloid a protein as a potential biomarker in predicting acute onset and association with in-hospital death in acute aortic dissection. BMC cardiovascular disorders. 2019;19:282.

[7] Ma C, Zhao H, Shi F, Li M, Liu X, Ji C, et al. Serum Ceruloplasmin Is the Candidate Predictive Biomarker for Acute Aortic Dissection and Is Related to Thrombosed False Lumen: a Propensity Score-Matched Observational Case-Control Study. Biological trace element research. 2021;199:895-911.

[8] Peng W, Peng Z, Chai X, Zhu Q, Yang G, Zhao Q, et al. Potential biomarkers for early diagnosis of acute aortic dissection. Heart & lung : the journal of critical care. 2015;44:205-8.

[9] Xiao Z, Xue Y, Yao C, Gu G, Zhang Y, Zhang J, et al. Acute Aortic Dissection Biomarkers Identified Using Isobaric Tags for Relative and Absolute Quantitation. BioMed research international. 2016;2016:6421451.

[10] Han C, Liu Q, Li Y, Zang W, Zhou J. S100A1 as a potential biomarker for the diagnosis of patients with acute aortic dissection. The Journal of international medical research. 2021;49:3000605211004512.

[11] Nagaoka K, Sadamatsu K, Yamawaki T, Shikada T, Sagara S, Ohe K, et al. Fibrinogen/fibrin degradation products in acute aortic dissection. Internal medicine (Tokyo, Japan). 2010;49:1943-7.

[12] Adem Çakır UP, Saliha Aksun, Ahmet Kayalı, Zeynep Karakaya, Fatih Esad Topal. Validity of Signal Peptide-CUB-EGF Domain-containing Protein-1 (SCUBE-1) in the Diagnosis of Aortic Dissection. Signa Vitae. 2020.

[13] Forrer A, Schoenrath F, Torzewski M, Schmid J, Franke UFW, Göbel N, et al. Novel Blood Biomarkers for a Diagnostic Workup of Acute Aortic Dissection. Diagnostics (Basel, Switzerland). 2021;11.

[14] Li T, Jing JJ, Yang J, Sun LP, Gong YH, Xin SJ, et al. Serum levels of matrix metalloproteinase 9 and toll-like receptor 4 in acute aortic dissection: a case-control study. BMC cardiovascular disorders. 2018;18:219.

[15] Yang Y, Jiao X, Li L, Hu C, Zhang X, Pan L, et al. Increased Circulating Angiopoietin-Like Protein 8 Levels Are Associated with Thoracic Aortic Dissection and Higher Inflammatory Conditions. Cardiovascular drugs and therapy. 2020;34:65-77.

[16] Giachino F, Loiacono M, Lucchiari M, Manzo M, Battista S, Saglio E, et al. Rule out of acute aortic dissection with plasma matrix metalloproteinase 8 in the emergency department. Critical care (London, England). 2013;17:R33.

[17] Suzuki T, Distante A, Zizza A, Trimarchi S, Villani M, Salerno Uriarte JA, et al. Preliminary experience with the smooth muscle troponin-like protein, calponin, as a novel biomarker for diagnosing acute aortic dissection. European heart journal. 2008;29:1439-45.

[18] Suzuki T, Katoh H, Tsuchio Y, Hasegawa A, Kurabayashi M, Ohira A, et al. Diagnostic implications of elevated levels of smooth-muscle myosin heavy-chain protein in acute aortic dissection. The smooth muscle myosin heavy chain study. Annals of internal medicine. 2000;133:537-41.

[19] Icer MA, Gezmen-Karadag M. The multiple functions and mechanisms of osteopontin. Clinical biochemistry. 2018;59:17-24.

[20] Li JJ, Han M, Wen JK, Li AY. Osteopontin stimulates vascular smooth muscle cell migration by inducing FAK phosphorylation and ILK dephosphorylation. Biochemical and biophysical research communications. 2007;356:13-9.

[21] Gao H, Steffen MC, Ramos KS. Osteopontin regulates α-smooth muscle actin and calponin in vascular smooth muscle cells. Cell biology international. 2012;36:155-61.

[22] Ye S, Sun Y, Bie A, Zhou Y, Liu J, Liu Q. Influence of osteopontin short hairpin RNA on the proliferation and activity of rat vascular smooth muscle cells. Journal of Huazhong University of Science and Technology Medical sciences = Hua zhong ke ji da xue xue bao Yi xue Ying De wen ban = Huazhong keji daxue xuebao Yixue Yingdewen ban. 2009;29:144-9.

[23] Fan F, Zhou Q, Xu Z, Wang D. Osteopontin in the Pathogenesis of Aortic Dissection by the Enhancement of MMP Expressions. International heart journal. 2019;60:429-35.

[24] Jang MA, Lee SJ, Baek SE, Park SY, Choi YW, Kim CD. α-Iso-Cubebene Inhibits PDGF-Induced Vascular Smooth Muscle Cell Proliferation by Suppressing Osteopontin Expression. PloS one. 2017;12:e0170699.

[25] Apte SS. A disintegrin-like and metalloprotease (reprolysin-type) with thrombospondin type 1 motif (ADAMTS) superfamily: functions and mechanisms. The Journal of biological chemistry. 2009;284:31493-7.

[26] Mead TJ, Apte SS. ADAMTS proteins in human disorders. Matrix biology : journal of the International Society for Matrix Biology. 2018;71-72:225-39.

[27] Santamaria S, de Groot R. ADAMTS proteases in cardiovascular physiology and disease. Open biology. 2020;10:200333.

[28] Porter S, Clark IM, Kevorkian L, Edwards DR. The ADAMTS metalloproteinases. The Biochemical journal. 2005;386:15-27.

[29] Gao Y, Wu W, Yu C, Zhong F, Li G, Kong W, et al. A disintegrin and metalloproteinase with thrombospondin motif 1 (ADAMTS1) expression increases in acute aortic dissection. Science China Life sciences. 2016;59:59-67.

[30] Wang S, Liu Y, Zhao G, He L, Fu Y, Yu C, et al. Postnatal deficiency of ADAMTS1 ameliorates thoracic aortic aneurysm and dissection in mice. Experimental physiology. 2018;103:1717-31.

[31] Li M, Liu Q, Lei J, Wang X, Chen X, Ding Y. MiR-362-3p inhibits the proliferation and migration of vascular smooth muscle cells in atherosclerosis by targeting ADAMTS1. Biochemical and biophysical research communications. 2017;493:270-6.

[32] Qu Y, Zhang N. miR-365b-3p inhibits the cell proliferation and migration of human coronary artery smooth muscle cells by directly targeting ADAMTS1 in coronary atherosclerosis. Experimental and therapeutic medicine. 2018;16:4239-45.

[33] Jönsson-Rylander AC, Nilsson T, Fritsche-Danielson R, Hammarström A, Behrendt M, Andersson JO, et al. Role of ADAMTS-1 in atherosclerosis: remodeling of carotid artery, immunohistochemistry, and proteolysis of versican. Arteriosclerosis, thrombosis, and vascular biology. 2005;25:180-5.

[34] Gendron C, Kashiwagi M, Lim NH, Enghild JJ, Thøgersen IB, Hughes C, et al. Proteolytic activities of human ADAMTS-5: comparative studies with ADAMTS-4. The Journal of biological chemistry. 2007;282:18294-306.

[35] Fushimi K, Troeberg L, Nakamura H, Lim NH, Nagase H. Functional differences of the catalytic and non-catalytic domains in human ADAMTS-4 and ADAMTS-5 in aggrecanolytic activity. The Journal of biological chemistry. 2008;283:6706-16.

[36] Santamaria S, Yamamoto K, Teraz-Orosz A, Koch C, Apte SS, de Groot R, et al. Exosites in Hypervariable Loops of ADAMTS Spacer Domains control Substrate Recognition and Proteolysis. Scientific reports. 2019;9:10914.

[37] Melching LI, Fisher WD, Lee ER, Mort JS, Roughley PJ. The cleavage of biglycan by aggrecanases. Osteoarthritis and cartilage. 2006;14:1147-54.

[38] Ren P, Hughes M, Krishnamoorthy S, Zou S, Zhang L, Wu D, et al. Critical Role of ADAMTS-4 in the Development of Sporadic Aortic Aneurysm and Dissection in Mice. Scientific reports. 2017;7:12351.

[39] Ren P, Zhang L, Xu G, Palmero LC, Albini PT, Coselli JS, et al. ADAMTS-1 and ADAMTS-4 levels are elevated in thoracic aortic aneurysms and dissections. The Annals of thoracic surgery. 2013;95:570-7.

[40] Martínez-Martínez E, Miana M, Jurado-López R, Rousseau E, Rossignol P, Zannad F, et al. A role for soluble ST2 in vascular remodeling associated with obesity in rats. PloS one. 2013;8:e79176.

[41] Yamamoto M, Umebashi K, Tokito A, Imamura J, Jougasaki M. Interleukin-33 induces growth-regulated oncogene-α expression and secretion in human umbilical vein endothelial cells. American journal of physiology Regulatory, integrative and comparative physiology. 2017;313:R272-r9.

[42] Aoki S, Hayakawa M, Ozaki H, Takezako N, Obata H, Ibaraki N, et al. ST2 gene expression is proliferation-dependent and its ligand, IL-33, induces inflammatory reaction in endothelial cells. Molecular and cellular biochemistry. 2010;335:75-81.

[43] Pollheimer J, Bodin J, Sundnes O, Edelmann RJ, Skånland SS, Sponheim J, et al. Interleukin-33 drives a proinflammatory endothelial activation that selectively targets nonquiescent cells. Arteriosclerosis, thrombosis, and vascular biology. 2013;33:e47-55.

[44] Choi YS, Choi HJ, Min JK, Pyun BJ, Maeng YS, Park H, et al. Interleukin-33 induces angiogenesis and vascular permeability through ST2/TRAF6-mediated endothelial nitric oxide production. Blood. 2009;114:3117-26.

[45] Mueller T, Jaffe AS. Soluble ST2--analytical considerations. The American journal of cardiology. 2015;115:8b-21b.

[46] Cikach FS, Koch CD, Mead TJ, Galatioto J, Willard BB, Emerton KB, et al. Massive aggrecan and versican accumulation in thoracic aortic aneurysm and dissection. JCI insight. 2018;3.

[47] Schuchardt M, Prüfer N, Tu Y, Herrmann J, Hu XP, Chebli S, et al. Dysfunctional high-density lipoprotein activates toll-like receptors via serum amyloid A in vascular smooth muscle cells. Scientific reports. 2019;9:3421.

[48] Yu MH, Li X, Li Q, Mo SJ, Ni Y, Han F, et al. SAA1 increases NOX4/ROS production to promote LPS-induced inflammation in vascular smooth muscle cells through activating p38MAPK/NF-κB pathway. BMC molecular and cell biology. 2019;20:15.

[49] Zhang X, Chen J, Wang S. Serum Amyloid A Induces a Vascular Smooth Muscle Cell Phenotype Switch through the p38 MAPK Signaling Pathway. BioMed research international. 2017;2017:4941379.

[50] Kumon Y, Hosokawa T, Suehiro T, Ikeda Y, Sipe JD, Hashimoto K. Acute-phase, but not constitutive serum amyloid A (SAA) is chemotactic for cultured human aortic smooth muscle cells. Amyloid : the international journal of experimental and clinical investigation : the official journal of the International Society of Amyloidosis. 2002;9:237-41.

[51] Wang X, Chai H, Wang Z, Lin PH, Yao Q, Chen C. Serum amyloid A induces endothelial dysfunction in porcine coronary arteries and human coronary artery endothelial cells. American journal of physiology Heart and circulatory physiology. 2008;295:H2399-408.

[52] Fox PL, Mazumder B, Ehrenwald E, Mukhopadhyay CK. Ceruloplasmin and cardiovascular disease. Free radical biology & medicine. 2000;28:1735-44.

[53] Dadu RT, Dodge R, Nambi V, Virani SS, Hoogeveen RC, Smith NL, et al. Ceruloplasmin and heart failure in the Atherosclerosis Risk in Communities study. Circulation Heart failure. 2013;6:936-43.

[54] Kennedy DJ, Fan Y, Wu Y, Pepoy M, Hazen SL, Tang WH. Plasma ceruloplasmin, a regulator of nitric oxide activity, and incident cardiovascular risk in patients with CKD. Clinical journal of the American Society of Nephrology : CJASN. 2014;9:462-7.

[55] Panichi V, Taccola D, Rizza GM, Consani C, Migliori M, Filippi C, et al. Ceruloplasmin and acute phase protein levels are associated with cardiovascular disease in chronic dialysis patients. Journal of nephrology. 2004;17:715-20.

[56] Reunanen A, Knekt P, Aaran RK. Serum ceruloplasmin level and the risk of myocardial infarction and stroke. American journal of epidemiology. 1992;136:1082-90.

[57] Klipstein-Grobusch K, Grobbee DE, Koster JF, Lindemans J, Boeing H, Hofman A, et al. Serum caeruloplasmin as a coronary risk factor in the elderly: the Rotterdam Study. The British journal of nutrition. 1999;81:139-44.

[58] Shukla N, Maher J, Masters J, Angelini GD, Jeremy JY. Does oxidative stress change ceruloplasmin from a protective to a vasculopathic factor? Atherosclerosis. 2006;187:238-50.

[59] Samet JM, Graves LM, Quay J, Dailey LA, Devlin RB, Ghio AJ, et al. Activation of MAPKs in human bronchial epithelial cells exposed to metals. The American journal of physiology. 1998;275:L551-8.

[60] Qian Q, Li M, Cai Y, Ward CJ, Somlo S, Harris PC, et al. Analysis of the polycystins in aortic vascular smooth muscle cells. Journal of the American Society of Nephrology : JASN. 2003;14:2280-7.

[61] Boulter C, Mulroy S, Webb S, Fleming S, Brindle K, Sandford R. Cardiovascular, skeletal, and renal defects in mice with a targeted disruption of the Pkd1 gene. Proceedings of the National Academy of Sciences of the United States of America. 2001;98:12174-9.

[62] Feng J, Ge S, Zhang L, Che H, Liang C. Aortic dissection is associated with reduced polycystin-1 expression, an abnormality that leads to increased ERK phosphorylation in vascular smooth muscle cells. European journal of histochemistry : EJH. 2016;60:2711.

[63] Zhang J, Liu F, He YB, Zhang W, Ma WR, Xing J, et al. Polycystin-1 Downregulation Induced Vascular Smooth Muscle Cells Phenotypic Alteration and Extracellular Matrix Remodeling in Thoracic Aortic Dissection. Frontiers in physiology. 2020;11:548055.

[64] Geissmann F, Manz MG, Jung S, Sieweke MH, Merad M, Ley K. Development of monocytes, macrophages, and dendritic cells. Science (New York, NY). 2010;327:656-61.

[65] Trakaki A, Marsche G. Current Understanding of the Immunomodulatory Activities of High-Density Lipoproteins. Biomedicines. 2021;9.

[66] Murphy AJ, Woollard KJ, Hoang A, Mukhamedova N, Stirzaker RA, McCormick SP, et al. High-density lipoprotein reduces the human monocyte inflammatory response. Arteriosclerosis, thrombosis, and vascular biology. 2008;28:2071-7.

[67] Catapano AL, Pirillo A, Bonacina F, Norata GD. HDL in innate and adaptive immunity. Cardiovascular research. 2014;103:372-83.

[68] Yu BL, Wang SH, Peng DQ, Zhao SP. HDL and immunomodulation: an emerging role of HDL against atherosclerosis. Immunology and cell biology. 2010;88:285-90.

[69] Feig JE, Rong JX, Shamir R, Sanson M, Vengrenyuk Y, Liu J, et al. HDL promotes rapid atherosclerosis regression in mice and alters inflammatory properties of plaque monocyte-derived cells. Proceedings of the National Academy of Sciences of the United States of America. 2011;108:7166-71.

[70] Huang X, Yue Z, Wu J, Chen J, Wang S, Wu J, et al. MicroRNA-21 Knockout Exacerbates Angiotensin II-Induced Thoracic Aortic Aneurysm and Dissection in Mice With Abnormal Transforming Growth Factor-β-SMAD3 Signaling. Arteriosclerosis, thrombosis, and vascular biology. 2018;38:1086-101.

[71] Wang Y, Dong CQ, Peng GY, Huang HY, Yu YS, Ji ZC, et al. MicroRNA-134-5p Regulates Media Degeneration through Inhibiting VSMC Phenotypic Switch and Migration in Thoracic Aortic Dissection. Molecular therapy Nucleic acids. 2019;16:284-94.

[72] Li T, Liu C, Liu L, Xia H, Xiao Y, Wang X, et al. Regulatory Mechanism of MicroRNA-145 in the Pathogenesis of Acute Aortic Dissection. Yonsei medical journal. 2019;60:352-9.

[73] Liao M, Zou S, Bao Y, Jin J, Yang J, Liu Y, et al. Matrix metalloproteinases are regulated by MicroRNA 320 in macrophages and are associated with aortic dissection. Experimental cell research. 2018;370:98-102.

[74] Shen H, Lu S, Dong L, Xue Y, Yao C, Tong C, et al. hsa-miR-320d and hsa-miR-582, miRNA Biomarkers of Aortic Dissection, Regulate Apoptosis of Vascular Smooth Muscle Cells. Journal of cardiovascular pharmacology. 2018;71:275-82.

[75] Qi YF, Shu C, Xiao ZX, Luo MY, Fang K, Guo YY, et al. Post-Transcriptional Control of Tropoelastin in Aortic Smooth Muscle Cells Affects Aortic Dissection Onset. Molecules and cells. 2018;41:198-206.

[76] Xue L, Luo S, Ding H, Liu Y, Huang W, Fan X, et al. Upregulation of miR-146a-5p is associated with increased proliferation and migration of vascular smooth muscle cells in aortic dissection. Journal of clinical laboratory analysis. 2019;33:e22843.

[77] Zhang M, Wang Z. Downregulation of miR143/145 gene cluster expression promotes the aortic media degeneration process via the TGF-β1 signaling pathway. American journal of translational research. 2019;11:370-8.

[78] Wang W, Wang Y, Piao H, Li B, Zhu Z, Li D, et al. Bioinformatics Analysis Reveals MicroRNA-193a-3p Regulates ACTG2 to Control Phenotype Switch in Human Vascular Smooth Muscle Cells. Frontiers in genetics. 2020;11:572707.

[79] Xiao Y, Sun Y, Ma X, Wang C, Zhang L, Wang J, et al. MicroRNA-22 Inhibits the Apoptosis of Vascular Smooth Muscle Cell by Targeting p38MAPKα in Vascular Remodeling of Aortic Dissection. Molecular therapy Nucleic acids. 2020;22:1051-62.

[80] Wang Z, Zhuang X, Chen B, Feng D, Li G, Wei M. The Role of miR-107 as a Potential Biomarker and Cellular Factor for Acute Aortic Dissection. DNA and cell biology. 2020;39:1895-906.

[81] Yang P, Wu P, Liu X, Feng J, Zheng S, Wang Y, et al. MiR-26b Suppresses the Development of Stanford Type A Aortic Dissection by Regulating HMGA2 and TGF-β/Smad3 Signaling Pathway. Annals of thoracic and cardiovascular surgery : official journal of the Association of Thoracic and Cardiovascular Surgeons of Asia. 2020;26:140-50.

[82] Sun Y, Xiao Y, Sun H, Zhao Z, Zhu J, Zhang L, et al. miR-27a regulates vascular remodeling by targeting endothelial cells' apoptosis and interaction with vascular smooth muscle cells in aortic dissection. Theranostics. 2019;9:7961-75.

[83] Tang Y, Yu S, Liu Y, Zhang J, Han L, Xu Z. MicroRNA-124 controls human vascular smooth muscle cell phenotypic switch via Sp1. American journal of physiology Heart and circulatory physiology. 2017;313:H641-h9.

[84] Zhao X, Cheng S, Li S, Li J, Bai X, Xi J. CDKN2B-AS1 Aggravates the Pathogenesis of Human Thoracic Aortic Dissection by Sponge to miR-320d. Journal of cardiovascular pharmacology. 2020;76:592-601.

[85] Zhang X, Wu H, Mai C, Qi Y. Long Noncoding RNA XIST/miR-17/PTEN Axis Modulates the Proliferation and Apoptosis of Vascular Smooth Muscle Cells to Affect Stanford Type A Aortic Dissection. Journal of cardiovascular pharmacology. 2020;76:53-62.

[86] Sun J, Chen G, Jing Y, He X, Dong J, Zheng J, et al. LncRNA Expression Profile of Human Thoracic Aortic Dissection by High-Throughput Sequencing. Cellular physiology and biochemistry : international journal of experimental cellular physiology, biochemistry, and pharmacology. 2018;46:1027-41.

[87] Lai Y, Li J, Zhong L, He X, Si X, Sun Y, et al. The pseudogene PTENP1 regulates smooth muscle cells as a competing endogenous RNA. Clinical science (London, England : 1979). 2019;133:1439-55.

[88] Wang W, Liu Q, Wang Y, Piao H, Zhu Z, Li D, et al. LINC01278 Sponges miR-500b-5p to Regulate the Expression of ACTG2 to Control Phenotypic Switching in Human Vascular Smooth Muscle Cells During Aortic Dissection. Journal of the American Heart Association. 2021;10:e018062.

[89] Wang P, Wang Z, Zhang M, Wu Q, Shi F. Lnc-OIP5-AS1 exacerbates aorta wall injury during the development of aortic dissection through upregulating TUB via sponging miR-143-3p. Life sciences. 2021;271:119199.

[90] Ren M, Wang T, Wei X, Wang Y, Ouyang C, Xie Y, et al. LncRNA H19 regulates smooth muscle cell functions and participates in the development of aortic dissection through sponging miR-193b-3p. Bioscience reports. 2021;41.

[91] Li S, Zhao X, Cheng S, Li J, Bai X, Meng X. Downregulating long non-coding RNA PVT1 expression inhibited the viability, migration and phenotypic switch of PDGF-BB-treated human aortic smooth muscle cells via targeting miR-27b-3p. Human cell. 2021;34:335-48.

[92] Li Y, Yang N, Zhou X, Bian X, Qiu G, Zhang M, et al. LncRNA and mRNA interaction study based on transcriptome profiles reveals potential core genes in the pathogenesis of human thoracic aortic dissection. Molecular medicine reports. 2018;18:3167-76.

[93] Tian C, Tang X, Zhu X, Zhou Q, Guo Y, Zhao R, et al. Expression profiles of circRNAs and the potential diagnostic value of serum circMARK3 in human acute Stanford type A aortic dissection. PloS one. 2019;14:e0219013.

[94] Zou M, Huang C, Li X, He X, Chen Y, Liao W, et al. Circular RNA expression profile and potential function of hsa_circRNA_101238 in human thoracic aortic dissection. Oncotarget. 2017;8:81825-37.

[95] Sodeck G, Domanovits H, Schillinger M, Ehrlich MP, Endler G, Herkner H, et al. D-dimer in ruling out acute aortic dissection: a systematic review and prospective cohort study. European heart journal. 2007;28:3067-75.

[96] Marill KA. Serum D-dimer is a sensitive test for the detection of acute aortic dissection: a pooled meta-analysis. The Journal of emergency medicine. 2008;34:367-76.

[97] Shimony A, Filion KB, Mottillo S, Dourian T, Eisenberg MJ. Meta-analysis of usefulness of d-dimer to diagnose acute aortic dissection. The American journal of cardiology. 2011;107:1227-34.

[98] Cui JS, Jing ZP, Zhuang SJ, Qi SH, Li L, Zhou JW, et al. D-dimer as a biomarker for acute aortic dissection: a systematic review and meta-analysis. Medicine. 2015;94:e471.

[99] Asha SE, Miers JW. A Systematic Review and Meta-analysis of D-dimer as a Rule-out Test for Suspected Acute Aortic Dissection. Annals of emergency medicine. 2015;66:368-78.

[100] Watanabe H, Horita N, Shibata Y, Minegishi S, Ota E, Kaneko T. Diagnostic test accuracy of D-dimer for acute aortic syndrome: systematic review and meta-analysis of 22 studies with 5000 subjects. Scientific reports. 2016;6:26893.
